# New chemical and microbial perspectives on vitamin B1 and vitamer dynamics of a coastal system

**DOI:** 10.1093/ismeco/ycad016

**Published:** 2024-01-10

**Authors:** Meriel J Bittner, Catherine C Bannon, Elden Rowland, John Sundh, Erin M Bertrand, Anders F Andersson, Ryan W Paerl, Lasse Riemann

**Affiliations:** Marine Biological Section, Department of Biology, University of Copenhagen, 3000 Helsingør, Denmark; Department of Biology, Dalhousie University, Halifax, B3H 4R2, Nova Scotia, Canada; Department of Biology, Dalhousie University, Halifax, B3H 4R2, Nova Scotia, Canada; Department of Biochemistry and Biophysics, National Bioinformatics Infrastructure Sweden, Science for Life Laboratory, Stockholm University, Box 1031, 17121 Solna, Sweden; Department of Biology, Dalhousie University, Halifax, B3H 4R2, Nova Scotia, Canada; Department of Gene Technology, Science for Life Laboratory, School of Engineering Sciences in Chemistry, Biotechnology and Health, KTH Royal Institute of Technology, 17165 Stockholm, Sweden; Department of Marine, Earth and Atmospheric Sciences, North Carolina State University, Raleigh, NC 2769, United States; Marine Biological Section, Department of Biology, University of Copenhagen, 3000 Helsingør, Denmark

**Keywords:** marine microbiology, vitamin B1, vitamin, thiamin, LC/MS, metagenomics, bacterioplankton, auxotrophy

## Abstract

Vitamin B1 (thiamin, B1) is an essential micronutrient for cells, yet intriguingly in aquatic systems most bacterioplankton are unable to synthesize it *de novo* (auxotrophy), requiring an exogenous source. Cycling of this valuable metabolite in aquatic systems has not been fully investigated and vitamers (B1-related compounds) have only begun to be measured and incorporated into the B1 cycle. Here, we identify potential key producers and consumers of B1 and gain new insights into the dynamics of B1 cycling through measurements of B1 and vitamers (HMP: 4-amino-5-hydroxymethyl-2-methylpyrimidine, HET: 4-methyl-5-thiazoleethanol, FAMP: *N*-formyl-4-amino-5-aminomethyl-2-methylpyrimidine) in the particulate and dissolved pool in a temperate coastal system. Dissolved B1 was not the primary limiting nutrient for bacterial production and was relatively stable across seasons with concentrations ranging from 74–117 pM, indicating a balance of supply and demand. However, vitamer concentration changed markedly with season as did transcripts related to vitamer salvage and transport suggesting use of vitamers by certain bacterioplankton, e.g. *Pelagibacterales*. Genomic and transcriptomic analyses showed that up to 78% of the bacterioplankton taxa were B1 auxotrophs. Notably, *de novo* B1 production was restricted to a few abundant bacterioplankton (e.g. *Vulcanococcus*, BACL14 (*Burkholderiales*), *Verrucomicrobiales*) across seasons. In summer, abundant picocyanobacteria were important putative B1 sources, based on transcriptional activity, leading to an increase in the B1 pool. Our results provide a new dynamic view of the players and processes involved in B1 cycling over time in coastal waters, and identify specific priority populations and processes for future study.

## Introduction

Vitamin B1 (B1 herein) is an essential micronutrient for all domains of life and primarily functions as an enzyme cofactor in a range of central metabolic processes [1, 2]. Select taxa synthesize B1 *de novo* (B1 prototrophs), a process often regulated by riboswitches and intracellular B1 concentration [3, 4]. However, other taxa cannot synthesize B1 *de novo* (B1 auxotrophs) and must acquire exogenous B1 or vitamers (B1-related compounds, including biosynthesis precursors and degradation products) to survive. HMP (4-amino-5-hydroxymethyl-2-methylpyrimidine), AmMP (4-amino-5-aminomethyl-2-methylpyrimidine), and FAMP (*N*-formyl-4-amino-5-aminomethyl-2-methylpyrimidine) are vitamers that can be salvaged for the pyrimidine part of the B1 molecule, whereas cHET (5-(2-hydroxyethyl)-4-methyl-1,3-thiazole-2-carboxylic acid) and HET (4-methyl-5-thiazoleethanol) are vitamers related to the thiazole part of the B1 molecule. While HMP and cHET can be intermediate compounds during the synthesis of B1, AmMP, FAMP, and HET are considered degradation products [1, 5, 6].

B1 auxotrophy in marine plankton was first recognized via cultivation experiments [7–10]. Since then genomic screening has been conducted and suggests B1 auxotrophy is prevalent in bacterio- and phytoplankton [2, 11–14]. Interestingly, B1 concentrations are generally low (picomolar) [15–17], and in some cases, B1 availability can limit bacterial production [11, 18] and phytoplankton biomass [18, 19]. As a result, B1/vitamer availability may regulate plankton dynamics and fluxes of biochemically important elements in marine systems [20, 21].

Dissolved B1 measured by mass spectrometry at coastal and open ocean sites ranges from below the detection limit of 0.81 pM to >500 pM [17, 22]. In coastal surface waters, concentrations <30 pM [5, 22, 23] as well as >100 pM have been reported [19, 24, 25]. Until recently, primarily concentrations for the vitamer HMP were available [15, 26, 27], but following recognition of other vitamers supporting marine plankton additional vitamer measurements have been made [5, 23, 28]. Recent vitamer data suggest they too are low in concentration (<30 pM), but the data are sparse and more measurements are necessary to elucidate broad environmental patterns as well as change relative to biochemistry and community structure. Microbial requirements for B1 are available for select plankton cultures and in the range of a few hundred to a few thousand molecules of B1 per cell [11, 12, 23, 27, 29, 30].

Taxa can use exogenous vitamers to synthesize intact B1 yet prototrophic cells are sources of “new” B1 through *de novo* synthesis. Bacterioplankton play key roles in marine B1-cycling, serving as sources, consumers [16, 29], and B1 transformers [2, 5, 29, 31] of B1/vitamers. For example, Cyanobacteria are prototrophs [2], putatively—but not yet definitively—serving as B1/vitamer sources to co-occurring plankton. Abundant SAR11 (*Pelagibacterales*) and SAR86 clade members are auxotrophs [13, 32], while Flavobacteria contain both prototrophs and auxotrophs [11, 28, 33, 34], indicating that population-specific information may be required to resolve taxa as B1 sources or sinks.

B1/vitamer transfer between marine plankton has been documented in laboratory experiments [9, 14, 35–37] and is presumed *in situ*. Few studies have investigated this through tracking dynamics in B1/vitamers and populations, and here insights have been hampered by limited taxonomic resolution of plankton communities [16, 22], assumptions about B1 genotypes [6, 28], or challenges measuring B1 [31, 33].

Measuring B1/vitamers by mass spectrometry is expensive, time consuming, and difficult due to their low (picomolar) concentrations in seawater and high water solubility [27, 38]. Accordingly, seawater measurements of B1 [16, 17, 19, 22] and vitamers [5, 15, 23, 28] are scarce. Nonetheless, dissolved and particulate measurements are critical for building complete B1 budgets and gaging the amount of vitamin/vitamer available to support bacterioplankton performing key biogeochemistry, as well as microbial food webs and productivity [39]. Combining chemical measurements with change in genes tied to B1 metabolism also has the potential to help identify key producers, consumers, and cycling occurring *in situ*.

Here, we sought to better understand the connectivity between B1/vitamer concentrations and genetic patterns of B1 synthesis, transport, and salvage within coastal microbial communities. To accomplish this, we simultaneously assessed B1/vitamer concentrations, microbial community composition, bacterioplankton B1 genotypes, and transcription profiles from spring to fall in a coastal fjord.

## Materials and methods

### Sampling and environmental parameters

Surface water from Roskilde fjord (RF), a 40-km long, shallow, and eutrophic estuary [40], was collected at monitoring station ROS60 (55° 42.00’ N, 12° 04.46′ E, Supplementary Fig. S1), where the 4.8-m water column is typically well mixed. Samples were collected by Niskin bottle via R/V Capella between 9 and 12:00 from March to November (except April) in 2020. Water was passed through a 90-μm mesh filter (except samples for chlorophyll *a* and phytoplankton analysis) to exclude larger planktonic organisms and collected in acid-washed bottles. Simultaneously, CTD profiles, Secchi depth, and biological/chemical parameters (chlorophyll *a*, inorganic nutrients, phytoplankton) were obtained by the Nature Agency, Denmark. Phytoplankton were microscopically identified and measured by the Utermöhl method [41], and biomass was calculated using taxa-specific conversion factors [41]. Bacterial production measurements were initiated immediately, and filtration for RNA extractions was complete within 2 h of sampling. Two bottle (2 L) amendment experiments tested for B1/vitamer limitation of the microbial community by comparing bacterial production measurements of a B1 (1 nM) and a vitamer (1 nM HMP plus 1 nM HET) amendment to a control treatment (Supplementary Methods 1).

NH_4_, NO_3_^−^ + NO_2_^−^, and PO_4_^3−^ concentrations were determined by a Lachat FIA-system [42] with detection limits of 0.214 μM, 0.071 μM, and 0.032 μM, respectively. Ethanol-extracted Chl *a* concentration was determined by UV-1800 UV/VIS spectrophotometer (Shimadzu). Particulate organic carbon (POC) was quantified using a TOC-5000A (Shimadzu; Supplementary Methods 2). Bacterioplankton counts were determined using SYBR Green I staining and flow cytometry (10 min, 0.5% EM-grade glutaraldehyde Sigma; frozen −80°C) [43]. Bacterial biomass was estimated based on flow cytometry cell counts and a conversion factor of 20 fg C per cell [44]. Bacterial production was determined by ^3^H-leucine incorporation (200 nM; l-[4,5-3H]-Leucine, 180 Ci mmol^−1^, Perkin Elmer; Supplementary Methods 3) [45].

### Vitamin B1 sample collection and analysis

Dissolved (<0.22 μm) and particulate (0.22–90 μm) B1 and vitamer concentrations were measured (full details in Supplementary Methods 4). All equipment was cleaned with methanol, and samples were processed in the dark. Three to five replicate 500-ml samples were filtered (<0.17 mbar) onto 47-mm nylon filters (0.22 μm; GVS North America) and frozen immediately (−80°C) for particulate vitamin analysis. Filtrates were stored in amber HDPE bottles at −20°C until processing.

B1 and vitamers were extracted from particulates on filters with use of bead beating and drying [46]. Dissolved B1 and vitamers were quantified via solid-phase extraction C18-cartridge capture [17, 27]. B1/vitamers were quantified using a Dionex Ultimate-3000 LC system coupled to the electrospray ionization source of a TSQ Quantiva triple-stage quadrupole mass spectrometer (ThermoFisher Scientific). Transition list and details are in Supplementary Table S1 and Supplementary Methods 4. Concentrations of metabolites were determined by calibration curves run in the matrix of a quality control sample, consisting of equal volumes of each sample. B1 peak area was normalized to ^13^C-B1. Dissolved B1 concentrations were corrected with sample-specific recovery of the internal ^13^C-B1 standard. Recovery of the internal standard ^13^C-B1 in the dissolved samples was 24 ± 7%. Estimates of dissolved B1 percent recovery vary (e.g. 55 ± 29 [38]), likely due to sample processing, detection method, internal standard preparation, recovery calculation and calibration curves [5, 17, 27, 38, 47]. Concentrations of dissolved vitamers were corrected for recovery with values obtained from Paerl et al. [5].

Limits of quantitation (LOQ) and limits of detection (LOD) were calculated as 10× and 3× the variation in the intersample blanks for all compounds (Supplementary Table S1). For dissolved samples, the LOD was calculated as the response variation in the quality control sample rather than the inter-sample blanks (Supplementary Table S1). Chromatograms for samples with compound concentrations (B1, HMP, FAMP) between LOD and LOQ were visually inspected and analyzed batch per batch [48]. Each metabolite measurement is reported as the mean of two technical replicates and only if the metabolite was above the LOD in both HPLC–MS injections.

B1, thiamin monophosphate (TMP) and vitamers HMP (4-amino-5-hydroxymethyl-2-methylpyrimidine), FAMP (N-formyl-4-amino-5-aminomethyl-2-methylpyrimidine), AmMP (4-amino-5-aminomethyl-2-methylpyrimidine), HET (4-methyl-5-thiazoleethanol), and cHET (5-(2-hydroxyethyl)-4-methyl-1,3-thiazole-2-carboxylic acid) were measured. Compound abbreviations are provided in Table 1A. Traces of AmMP, and cHET and TMP, were detected but mostly below LOQ and excluded from further analysis (Supplementary Table S1).

**Table 1:** Abbreviations for vitamin B1 and vitamers (A) and genes for thiamin-related enzymes (B) referred to in the text and figures.

**A**	**Abbreviated name**	**Chemical name**	**Category**
	B1	Thiamin	
	TMP	Thiamin monophosphate	
	TDP	Thiamin diphosphate	
	HET	4-Methyl-5-thiazoleethanol	Thiazole vitamer
	cHET	﻿5-(2-Hydroxyethyl)-4-methyl-1,3-thiazole-2-carboxylic acid	Thiazole vitamer
	HMP	4-amino-5-hydroxymethyl-2-methylpyrimidine	Pyrimidine vitamer
	AmMP	4-amino-5-aminomethyl-2-methylpyrimidine	Pyrimidine vitamer
	FAMP	*﻿N*-formyl-4-amino-5-aminomethyl-2-methylpyrimidine	Pyrimidine vitamer
**B**	**Gene**	**Encoded enzyme**	**Category**
	*thiC*	Phosphomethylpyrimidine synthase	Pyrimidine synthesis
	*thiG*	Thiazole biosynthesis protein	Thiazole synthesis
	*thiE*	Thiamin monophosphate synthase	B1 synthesis
	*thiB*	Thiamin-binding protein of ABC transporter (ThiBPQ)	B1 (/TMP/TDP) transport
	*thiT*	Energy-coupled thiamine transporter	B1 transport
	*thiY*	Pyrimidine precursor-binding protein of ABC transporter (ThiXYZ)	Pyrimidine(/B1) transport
	*thiV*	Sodium:solute symporter	Pyrimidine(/B1) transport
	*cytX*	Putative hydroxymethylpyrimidine transporter	Putative B1/pyrimidine transport
	*ykoF*	B1/HMP-binding protein	B1/HMP transport
	*tenA*	Thiaminase II	Pyrimidine salvage
	*thiM*	Thiazole kinase	Thiazole salvage
	*thiPerm*	Transporter of nucleobase or vitamin	Potential B1 transport
	*omr1*	TonB-dependent binding protein of putative transporter	Potential B1 transport

One bioassay with B1-auxotrophic *Vibrio anguillarum* PF430–3 *ΔthiE* was carried out to determine bioavailable dissolved B1 [11]. Filtered water (<0.2 μm) from 4 June 2020 was diluted 1:10 with Aquil medium without vitamin and metal solution [49] and supplemented with macro nutrients (glucose 100 μM, ammonium 40 μM, phosphate 2.5 μM). Four replicates were run in parallel for initial samples (no B1 addition), an internal standard curve (5, 10, 25, 50, 75 pM) and negative control tubes (medium with no B1 addition), incubated in the dark at 16°C on a shaker. PF430–3 abundances were determined by flow cytometry as described above for RF bacterioplankton.

### Nucleic acid sampling, extraction, and sequencing

For DNA and RNA extraction, 1 L of sample was gently filtered via peristaltic pump onto a 0.22-μm Sterivex filter (PES, Millipore). Filters for RNA extraction were preserved with RNAlater (Sigma-Aldrich). Filters were sealed and stored at −80°C until extraction. The filters were crushed by flash freezing and grinding. DNA was extracted with the DNeasy Blood and Tissue kit (Qiagen, Hilden, Germany) with additional lysozyme (Sigma-Aldrich) and proteinase K (Qiagen) treatments, and quantified (PicoGreen, Invitrogen). RNA was extracted with the RNeasy RNA Mini extraction kit (Qiagen), and the RNase-free DNase Set (Qiagen) was used to remove DNA. Additional DNase treatments (Turbo DNA-free kit, Invitrogen) were carried out followed by a column clean up with the RNA Clean and Concentrator-5 kit (Zymo Research, Freiburg, Germany). DNA removal was confirmed by 16S rRNA gene PCR [50].

The 16S and 18S rRNA genes were amplified using 515F-Y/926R primers [50] and KAPA HiFi HotStart ReadyMix (Roche; Supplementary Table S2). For each sample, triplicate PCR reactions were pooled and cleaned (Gene Clean kit, MP Biomedicals). Samples were indexed, purified (Agencourt XP Beads, Beckman Coulter), quantified, pooled in equimolar ratio, and sequenced with MiSeq 2 × 300 bp, v3 chemistry (Illumina) at the GeoGenetics Sequencing Core of the University of Copenhagen.

Metagenomic sequencing was carried out on a NovaSeq 6000 S4 (2 × 150 bp, Illumina) at the National Genomics Infrastructure in Stockholm, Sweden. The library was prepared using 15 ng DNA with the SMARTer Thruplex kit (Takara). Metatranscriptomic sequencing was conducted using NovaSeq 6000 S4 (2 × 150 bp, Illumina) at the National Genomics Infrastructure in Uppsala, Sweden. The library was prepared using 200 ng total RNA per sample in the Stranded Total RNA-Prep Ligation with Ribo-Zero Plus Kit (Illumina). Sequencing summary statistics are in Supplementary Table S3.

### Sequence and data analysis

Amplicon reads were processed for 16S and 18S rRNA gene sequence variants (ASVs) [51]. Reads were filtered, trimmed, dereplicated, denoised; read pairs were merged, and chimeras were removed in dada2 (v1.22.0, https://benjjneb.github.io/dada2/index.html, Supplementary Methods 5.1). Taxonomy was assigned to 16S and 18S rRNA gene ASVs with “assignTaxonomy” from dada2 with the SBDI-curated version (v5) [52] of 16S sequences of GTDB (r07-rs207) [53], and with “IdTaxa” from DECIPHER [54] with the PR2 (v4.13.0) [55] database, respectively. Further processing was carried out using phyloseq (v1.38.0) [56].

Data from 17 metagenomic (12 main samples, 5 additional samples, Supplementary Table S4) and 12 metatranscriptomic samples (corresponding to the 12 main metagenomic samples) was processed with a Snakemake pipeline (https://github.com/EnvGen/B1-Ocean) based on the nbis-meta workflow (https://github.com/NBISweden/nbis-meta) (full details in Supplementary Methods 5.2). In brief, assemblies were generated with MEGAHIT (v1.2.9) [57] (17 single and 3 coassemblies, Supplementary Table S4). For predicted genes, hmmsearch (v3.3.2, http://hmmer.org) was run with the Pfam database (v31.0) [58], and additional HMM profiles (B1-related biosynthesis, salvage and transporter proteins) were obtained from TIGRFAM (v15.0) [59] and Paerl et al. [11] (Supplementary Table S5). Protein annotations related to B1 were further verified manually and if necessary filtered by a custom cutoff (Supplementary Methods 5.3). Additionally, thiamin diphosphate riboswitches (THI-box) were identified on contigs with the TDP riboswitch model (TPP, RF00059) with infernal [4, 60]. Assembled contigs were binned with MetaBAT2 (v2.14) [61], quality assessed with CheckM (v1.1.2) [62], phylogenetically classified with GTDB-Tk (v2.1.0) [63], and average nucleotide identity (ANI) was calculated with fastANI (v1.3) [64]. Bins considered at least medium-quality draft metagenome-assembled genomes (MAGs; ≥50% completion, <10% contamination) [65] were clustered at 95% ANI [66].

Protein coding genes (Table 1B) were analyzed on two levels to provide an understanding of B1 physiology (i) on contigs on assembly level and (ii) in MAG clusters, based on all MAGs per cluster. For gene abundances on the assembly level, values were normalized to Reads Per Kilobase Million mapped reads (RPKM, reads that were assigned to ORFs in the metagenomic assembly) and then to selected single-copy marker gene abundances (Supplementary Table S6).

Metatranscriptomic samples were processed with FastQC and trimmomatic (v0.39) [67] for quality and adapter trimming. To remove rRNA reads, sortMeRNA (v2.1b, all rRNA databases) [68] was run before mapping metatranscriptomic reads to the annotated metagenomic assemblies with bowtie2 (v2.4.5) [69]. Transcript counts were summed for each protein annotation, followed by RPKM normalization. At the assembly level, values were divided by the median RPKM counts of selected single-copy marker genes (Supplementary Table S6) and expressed as relative values per assembly [70]. For hmm models, 21 Pfam models were used for normalizing the hmmsearch counts (Supplementary Table S6).

Amplicon sequences and output files from the metagenomic and metatranscriptomic workflow were analyzed and visualized in the R environment (v4.1.3) [71] with tidyverse (v1.3.1) [72]. Heatmaps were generated with pheatmap [73]. Kendall correlation analyses were conducted with rstatix (v0.7.0, A. Kassambara: https://github.com/kassambara/rstatix), and correlations (τ) with a *p* < .05 were considered significant (Supplementary Table S7).

## Results

### Environmental conditions during sampling

**Figure 1:**
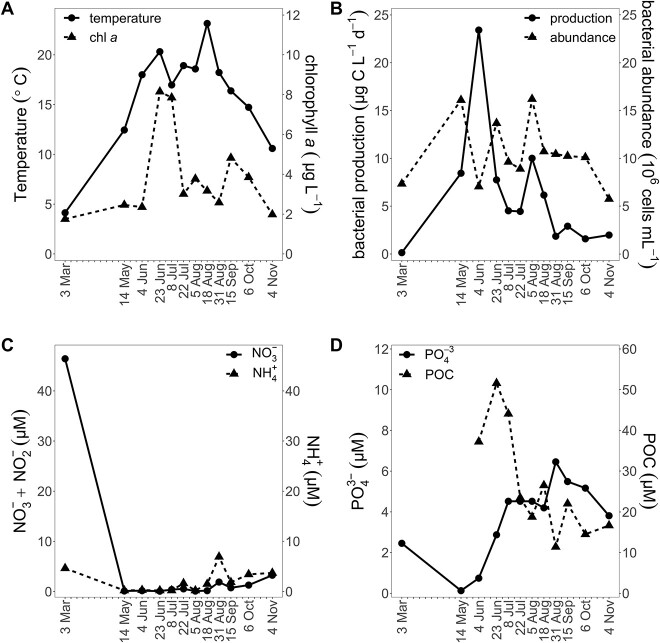
Seasonal changes in abiotic and biotic parameters in 2020. Temperature and chlorophyll *a* (Chl *a*) (A), bacterial production and abundance (B), concentrations of ammonia and nitrate/nitrite (C), and phosphate and POC (D).

In the surface water, temperature ranged from 4.1 to 23.1°C (Fig. 1A), salinity from 11.57 to 14.28, and maximum Chl *a* concentrations were in June/July (8.15/7.85 μg l^−1^). Several phytoplankton blooms occurred during the year depleting inorganic nutrients (Fig. 1A, C, and D). Based on the nutrient dynamics, we assume that a phytoplankton bloom occurred during late March/April when sampling was not possible (Fig. 1C and D). Bacterial abundance ranged from 5.75 to 16.20 × 10^6^ ml^−1^ with peaks in May and August. Bacterial production (0.14–23.42 μg C l^−1^ d^−1^) showed two maxima (June and August) and was not enhanced in the two B1/vitamer amendment experiments (June, August; Supplementary Fig. S2).

### Vitamin B1 and vitamers concentrations and correlations

**Figure 2:**
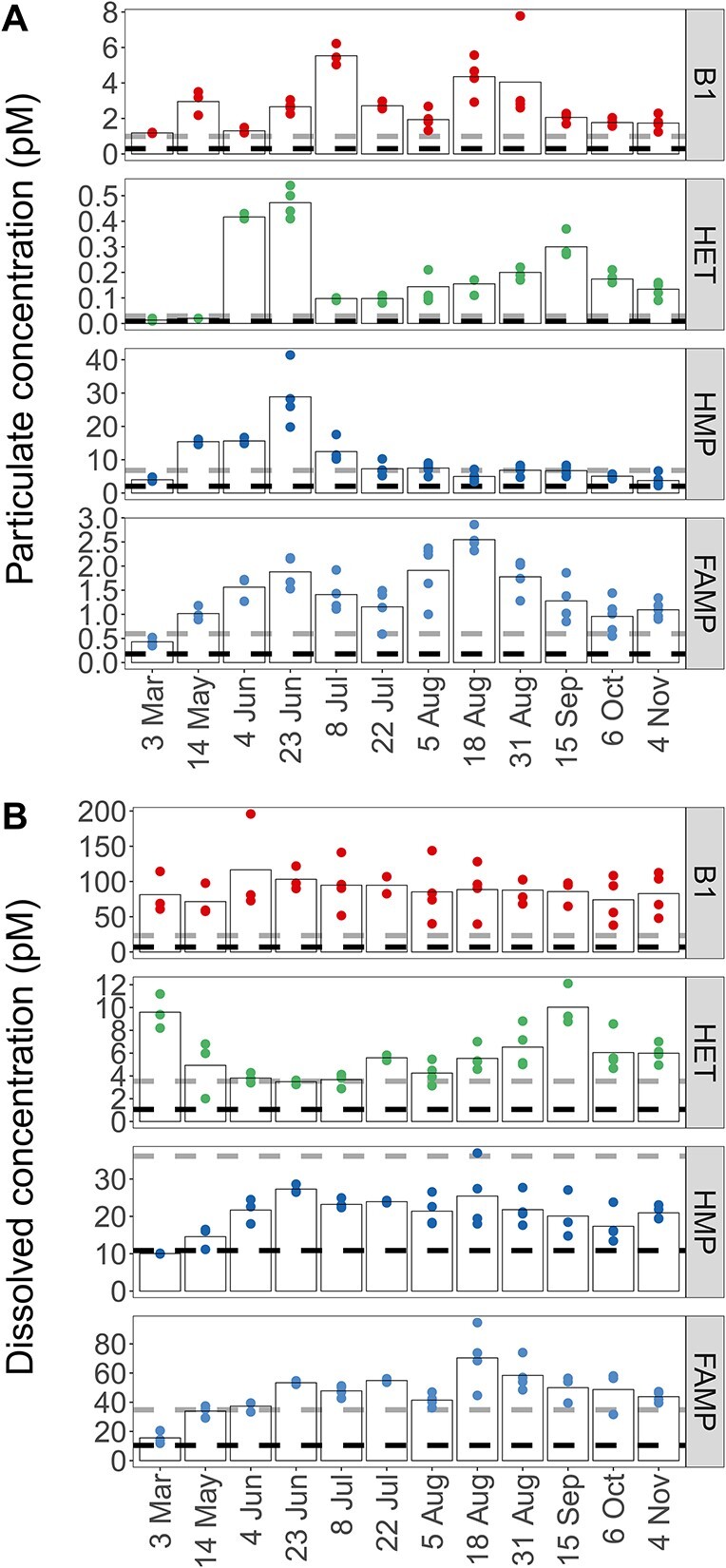
Concentrations of particulate (A) and dissolved (B) B1 and vitamers (HET, HMP, FAMP) across seasons; black and gray dashed lines indicate LOD and quantification, respectively; biological replicates are shown for each time point. Bars indicate the mean of the biological replicates; one measurement of dissolved FAMP from 6 October was removed as an outlier; for compound abbreviations, see Table 1A; particulate values normalized to particulate organic carbon are provided in Supplementary Fig. S5; dissolved measurements are corrected for percent recovery.

Concentrations of dissolved and particulate vitamin B1, HET, HMP, and FAMP were quantified (Fig. 2). The combined particulate pool of B1 and vitamers peaked on 23 June (33.9 pM), was lowest in March (5.6), and was positively correlated with bacterial production (τ = 0.52, Supplementary Fig. S3). The combined dissolved pool (sum of the mean concentrations of B1, HET, HMP, and FAMP) of the four measured compounds was also lowest in March (117 pM) but highest in October with 210 pM, after the particulate maximum.

Total B1 (dissolved and particulate) was relatively stable throughout the year with an average concentration of 92 pM (standard deviation ±12 pM). Particulate B1 ranged from 1.2 pM in March to 5.5 pM in the beginning of July (Fig. 2A). Dissolved vitamin B1 ranged from 72 to 117 pM with concentrations above 100 pM in early summer (May/June), and lower concentrations toward fall (Fig. 2B). Bioavailable dissolved B1 for 4 June was 126 ± 10.5 pM determined by a bioassay (Supplementary Fig. S4 and Table S8). In comparison, B1 concentration for this day based on LC–MS was 117 ± 68.8 pM.

Particulate HET concentrations were about 10× lower than B1 (0.01–0.47 pM), and dissolved HET concentrations ranged from 3.5 to 10.0 pM (Fig. 2B). Particulate HMP (3.8–28.9 pM) peaked in early summer with 28.9 pM (23 June, Fig. 2A) and was lowest in November (3.8 pM, Fig. 2A). Maximum dissolved HMP was on 23 June (27.3 pM), and concentrations were elevated from June to August, later in summer than particulate HMP (Fig. 2B). Overall, dissolved HMP ranged from 9.9 to 27.3 pM and was positively correlated to dissolved B1 (τ = 0.64). Dissolved HMP and HET were negatively correlated (τ = −0.45, Supplementary Fig. S3) and peaked in different seasons (Fig. 2B).

Particulate FAMP ranged from 0.43 to 2.55 pM with higher concentrations in summer (Fig. 2A). Dissolved FAMP ranged from 16 to 112 pM and was also highest in summer, similarly to the pyrimidine vitamer HMP (Fig. 2B). Dissolved FAMP was positively correlated to dissolved HMP (τ = 0.52). Particulate and dissolved FAMP were positively correlated with temperature (dissolved τ = 0.55, particulate τ = 0.73). Particulate values normalized to POC, a proxy for B1 or vitamer per biomass, showed highest B1/POC and vitamer/POC ratios at the end of August (Supplementary Fig. S5) at peak temperature (23.1°C).

### Bacterial and eukaryotic biomass and community composition

**Figure 3:**
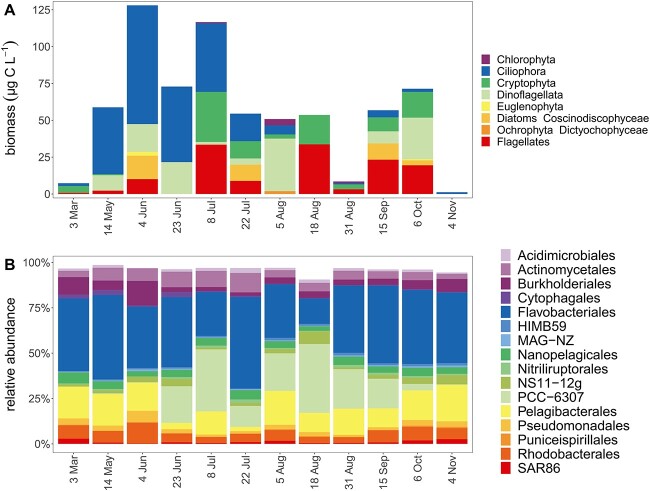
Dynamics of eukaryotes and bacterioplankton taxa across seasons. Eukaryotic biomass based on microscopy identification and enumeration (A); relative abundances of dominant bacterial orders (top 16) based on 16S rRNA gene amplicon sequencing (B).

*Ciliophora* dominated eukaryotic biomass (23.6 μg C l^−1^) followed by *Dinoflagellata* and flagellates (16.1 μg C l^−1^, 15.0 μg C l^−1^; Fig. 3A). *Dinoflagellata* and *Cryptophyta* were also detected among 18S rRNA ASVs (Supplementary Fig. S6). Highest eukaryotic biomass occurred in June/July coinciding with high POC concentrations (Fig. 1D). Diatoms, *Euglenophyta*, and *Chlorophyta* accounted for less than 10% of the eukaryotic biomass (Fig. 3A). Eukaryotic plankton biomass was only positively correlated with particulate HMP (τ = 0.58, Supplementary Fig. S3A).

Bacterial biomass exceeded eukaryotic biomass—210 μg C l^−1^ vs 57 μg C l^−1^ on average—based on microscopy, flow cytometry, and published conversion factors (Supplementary Table S9) [44], suggesting that a large fraction of B1/POC was within bacterioplankton biomass (Supplementary Fig. S5). The bacterial phyla *Bacteroidota*, *Proteobacteria*, *Cyanobacteria*, and *Actinobacteria* dominated the prokaryotic community based on relative abundance of reads of 16S rRNA gene ASVs (Fig. 3B). *Flavobacteriales* (*Bacteriodota*) were most abundant, accounting on average for 36% of reads. The picocyanobacterial order *PCC-6307* was abundant from late June, correlating with an increase in Chl *a*, to October contributing up to 38% of the reads in summer, and decreasing toward fall with declining water temperature (Figs. 1A, 3B). *PCC-6307* also correlated positively with particulate B1 concentrations (τ = 0.64, Fig. 2A), temperature (τ = 0.58, Fig. 1A), and Chl *a* (τ = 0.55, Fig. 1A).

### Temporal change in genes and transcripts related to vitamin B1 physiology

**Figure 4:**
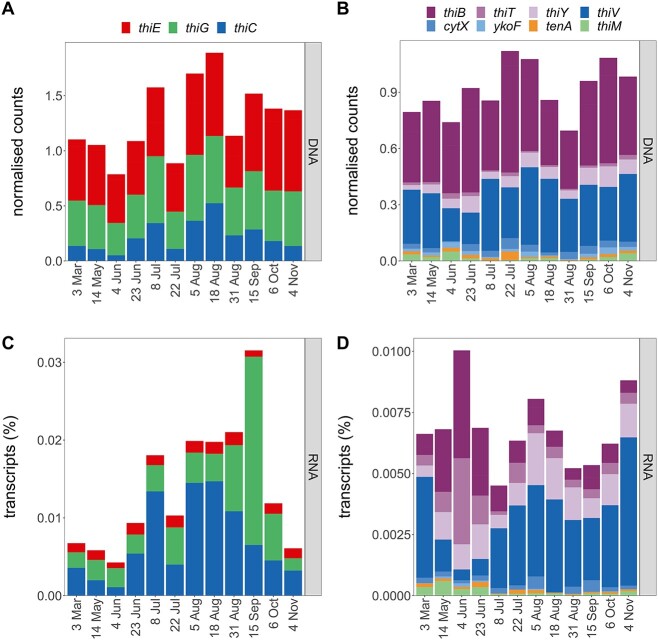
Temporal dynamics of genes and transcripts encoding proteins for B1 synthesis, transport, and salvage; normalized counts of B1 biosynthesis genes (A) and transcripts (C) and of gene and transcripts associated with B1 or vitamer transport or salvage (B, D); metagenomic counts are normalized by RPKM and by marker genes’ RPKM (A, B) and metatranscriptomic values to the normalized metagenomic counts and shown as relative transcripts per sample (C, D); for gene information, see Table 1B.

Twelve single assemblies, which had corresponding metatranscriptomes, were surveyed for genes encoding proteins for B1 synthesis, transport, and salvage (Fig. 4). B1 prototrophs canonically possess B1 synthesis genes *thiC*, *thiG*, and *thiE* at a ratio of 1:1:1; thus, a lower ratio on community level indicates a prevalence of auxotrophic populations. Gene numbers for *thiG* (thiazole synthase) and *thiE* (thiamin monophosphate synthase) were similar (0.9:1), while *thiC* (pyrimidine synthase) was low relative to *thiE* (0.64:1). No significant correlations between *de novo* synthesis gene counts and B1/vitamer concentrations were found except a weak negative correlation between *thiE* gene counts and particulate HMP (τ = −0.47, Supplementary Table S7).

Most B1 synthesis transcripts were accounted for by *thiE* and *thiC* (41% and 49%). Highest transcription of *thiG* coincided with high dissolved HET concentrations in September (Figs. 2 and 4C). Fewest *thiC* genes and transcripts (signaling prevalent auxotrophy) occurred on 4 June (Fig. 4C), and transcripts were positively correlated with relative abundance of Cyanobacteria (τ = 0.67).

Genes for B1 transporters (*thiB*, *thiT*) were present throughout the year with *thiB* genes being most abundant, and *thiB* and *thiT* transcript abundances were similar (Fig. 4B and D). Highest transcription of B1 transporters was on 4 June and coincided with the lowest *thiC* transcription and lowest relative abundances of prototrophic clusters (Supplementary Fig. S7). Putative transporters for pyrimidine vitamers (*thiV*, *thiY*, *cytX*, *ykoF*) were detected in DNA and RNA (Fig. 4B and D). Gene counts for *thiV*, a ﻿putative HMP sodium symporter [13], were comparable to *thiB*, but *thiV* transcripts were higher than other detected B1 transporters (*thiB*, *thiT*). Similar to *thiT*, transcription of *thiY*, coding for part of a HMP and/or B1 ABC transporter [74–76], was relatively high compared to its gene count.

Genes and transcripts for B1 salvage from pyrimidine (*tenA*) or thiazole (*thiM*) vitamers were present but in low numbers (Fig. 4B and D). While *tenA* transcripts were relatively stable over time, *thiM* transcripts were more prevalent from March to June.

### Metagenome-assembled genome generation, clustering, and abundances

**Figure 5:**
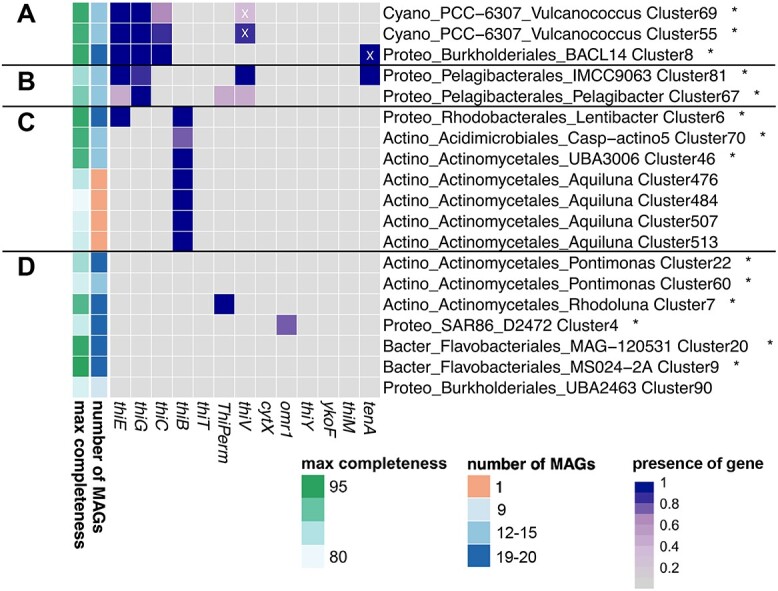
B1-related genotypes of the 19 most abundant clusters; heatmap shows presence (dark boxes) or absence (gray boxes) of proteins across clusters; the gradient of the dark boxes indicates the percentage of MAGs containing the protein. Number of MAGs and maximum estimated completeness of the MAGs are shown on the left side; taxonomy is shown for each cluster on phylum (Cyano: *Cyanobacteria*, Proteo: *Proteobacteria*, Actino: *Actinobacteriota*, Bacter: *Bacteroidota*), order and genus levels; relative abundances of clusters marked with asterisks are provided in Supplementary Fig. S10; prototrophic clusters with synthesis genes *thiE*, *thiG*, *thiC* (A), pyrimidine auxotrophic clusters with genes *thiE*, *thiG*, and transporter *thiV* (B), auxotrophic clusters capable of B1 transport with *thiB* (C), and auxotrophic clusters with putative transporters (D); white X’s indicate proteins that are considered false positives based on manual evaluation.

On average, 75% of the reads from 20 assemblies mapped to bins—indicating that bins represented most of the community (Supplementary Fig. S8). Of the 3982 bins obtained, 2180 were medium- or higher-quality MAGs forming 405 bacterial clusters (≥95% ANI; Supplementary Fig. S9). The dataset included 1025 MAGs with high completeness (≥90% completeness, <5% contamination) from 195 clusters. We established B1 genotypes of clusters by screening all MAGs in each cluster for B1-related genes. We investigated the putative B1 physiology of the 195 clusters containing minimum one high completeness MAG and the top 19 most abundant clusters (Fig. 5) in more detail. The top 19 clusters included the 14 most abundant clusters overall in the dataset across time points (Supplementary Fig. S10) and five additional clusters showing high relative abundance (among top two most abundant) at individual time points (e.g. Cluster 90).

### Vitamin B1 genotypes across microbial populations

Only 3 of the 19 abundant clusters were B1 prototrophic (possessing *thiC*, *thiG*, *thiE*; Fig. 5A). Two of the prototrophic clusters were picocyanobacterial (*Vulcanococcus*), and the third was Gammaproteobacterial (*Burkholderiales BACL14*). Clusters deficient in only one B1 synthesis gene were of the order *Pelagibacterales* (lack of *thiC*, Fig. 5B) and possessed the pyrimidine vitamer transporter gene *thiV*, adjacent to a TDP riboswitch and B1 salvage gene *tenA* (Cluster 81), as previously described for *Pelagibacterales* [13, 77]. Most *Actinobacteria* appeared reliant on exogenous dissolved B1 and transport via *thiB* to meet their need for intact B1 (Fig. 5C, Supplementary Fig. S11), while others possessed putative B1 transporters, *cytX*, or *ThiPerm*. Beyond *Actinobacteria*, the transporter *omr1* occurred in two SAR86 clusters (e.g. Cluster 4, Fig. 5D) and two *Flavobacteriales* clusters (e.g. Cluster 207, Supplementary Fig. S11) with a nearby TDP riboswitch.

Among the 195 clusters containing at least one high-quality MAG (≥90% completion; <5% contamination), only 22% were prototrophic (Supplementary Fig. S11). Several *Bacteroidota* and *Actinobacteriota* clusters lacked currently described synthesis and transport systems for B1 and vitamers (Fig. 5, Supplementary Fig. S11).

### Main *de novo* synthesizers of vitamin B1

**Figure 6:**
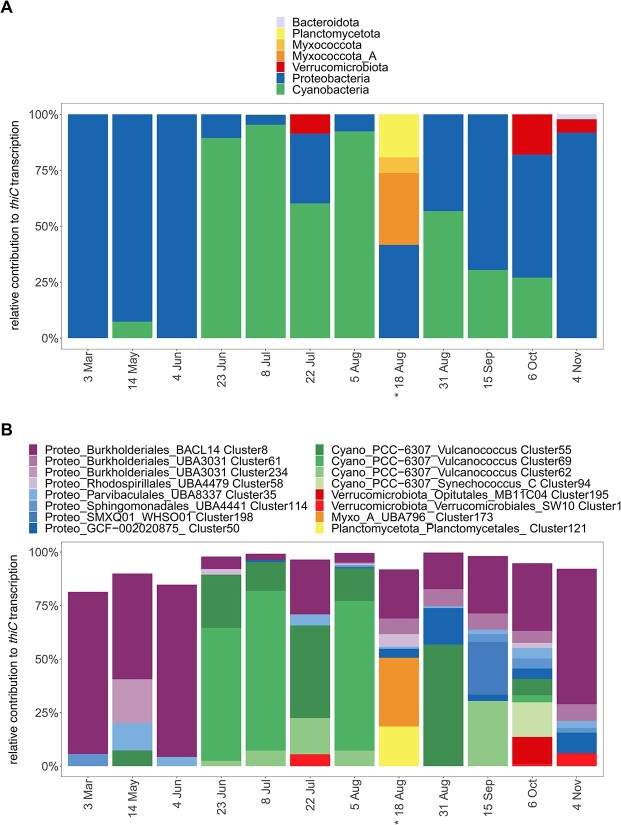
Transcription of *thiC* at the phylum and microbial population level. Relative contribution to *thiC* transcription levels of phyla (A) and the 16 clusters with highest *thiC* transcription (B); transcripts of *thiC* were selected as an indication of B1 *de novo* synthesis. Taxonomy of clusters is shown on phylum (Cyano: *Cyanobacteria*, Proteo: *Proteobacteria*, Myxo_A: *Myxococcota_A*), order and genus level; asterisks indicate that substantial *thiC* transcription occurred by two additional cyanobacteria bins that could not be clustered due to high strain heterogeneity; for each time point, total *thiC* transcription is scaled to 100%.

We focused on the transcriptional activity of microbial populations synthesizing B1 *de novo* as most populations in RF were auxotrophic, as is common in aquatic systems [11, 13, 22]. Transcription levels of the core genes for *de novo* synthesis were described above (Fig. 4C). We selected transcription of *thiC* as an indication of *de novo* synthesis as thiazole auxotrophs (lack of *thiG*) were rare (only detected in clusters with less than three MAGs and/or lower genome completion estimates). Of the 65 clusters encoding *thiC*, 53 were prototrophs (Supplementary Fig. S12). Here, we explored the contribution of the 65 clusters to *thiC* transcription over time. *Cyanobacteria* and *Proteobacteria* accounted for most *thiC* transcripts (Fig. 6A). At single time points, *Verrucomicrobiota* contributed with up to 18% of *thiC* transcripts in October (Fig. 6A, Supplementary Fig. S13).

From end of June to end of August, picocyanobacterial clusters (*Vulcanococcus*) contributed up to 95% of *thiC* transcription (Fig. 6), in line with their high relative abundances (Fig. 3B, Supplementary Fig. S6B) and overall high transcription. *Vulcanococcus* Clusters 69 and 55 were among the most abundant clusters (Fig. 5) and were the main B1 synthesizers during summer (23 June, 8 July, 5 August). As an exception, two *Vulcanococcus* bins displayed high *thiC* transcription on 18 August, but these were not assigned to clusters due to high estimated contamination (Supplementary Fig. S7A). This probably resulted from high strain heterogeneity of the bins, but both bins could be classified to the same species levels as Clusters 69 and 55.

Proteobacterial *thiC* transcription originated from 23 different clusters across different orders. *Burkholderiales* Cluster 8 showed continuous *thiC* transcription and Cluster 61 transcription in late summer and fall (Fig. 6B). When *Cyanobacteria* were absent, Cluster 8 *thiC* transcripts were dominant and contributed on average 34% of *thiC* transcription. Other Proteobacterial clusters that transcribed *thiC* were affiliated with the orders *SMXQ01*, *GCF-002020875*, *Parvibaculales*, *Rhodospirillales*, and *Sphingomonadales*.

## Discussion

### Environmental vitamin B1 and vitamer dynamics

While temporal variability in B1/vitamer concentrations has been addressed in a few studies [16, 19, 22, 25], the dynamics of B1/vitamers across particulate and dissolved pools in marine waters are basically unknown [15, 27], in contrast with well-described and modeled macronutrient dynamics [78–80]. The dissolved and particulate data obtained here provide several new insights that alter our view of B1 cycling in coastal waters.

First, dissolved B1 concentrations (mean 89 pM) were similar to those reported from other coastal environments using direct (LC–MS or HPLC) [5, 16, 25] or indirect (bioassay) measurements [11, 81, 82]. Dissolved B1 was notably stable across seasons—indicating tight coupling of supply and demand despite strong seasonal change in bacterioplankton and phytoplankton composition (Fig. 3). Similarly, a recent study found no seasonal differences in dissolved B1 in oligotrophic waters near the Bermuda Atlantic Time-Series Study site (BATS) [47]. Available B1 presumably helps plankton avoid B1-limitation; accordingly, we found that bacterial production was not stimulated in two B1 amendment experiments (Supplementary Fig. S2). Moreover, *Vibrio* bioassays yielded similar estimates of B1 to those determined by LC–MS, emphasizing the bioavailability of B1. B1 limitation of bacterioplankton has been rarely assessed—with some indication of primary or colimitation [11, 18, 31, 33], but our results indicate B1 is not primarily limiting bacterial production in RF. The prevalence of cornerstone B1 prototrophic populations, picocyanobacteria as well as select nonphotosynthetic bacteria (Supplementary Fig. S7), likely prevents B1 bacterioplankton limitation in RF. Considering systems beyond RF, an absence or reduced activity of these cornerstone B1 prototrophs may indicate regions where B1 is limiting bacterial growth, e.g. in polar and/or aphotic waters.

Second, vitamer concentrations changed seasonally and with distinct patterns depending on the vitamer (e.g. dissolved HMP and HET concentrations were negatively correlated, Fig. 2, Supplementary Fig. S3). Moreover, seasonal changes in vitamer concentrations were more pronounced compared to B1. Recent data from the open ocean BATS station also points to greater vitamer dynamics (specifically HET increasing in summer) in the surface ocean across seasons compared to B1 [47]. It appears in RF, and beyond, that vitamer supply and demand is more dynamic than that of B1 and that vitamer use is advantageous despite a higher abundance of B1.

Prior research suggests rivers and sediment may be sources of B1 and other B vitamins to coastal plankton [10, 83]; yet, we found no clear evidence of this in RF based on correlation analyses including B1/vitamer data and environmental data (especially salinity and Secchi depth). Positive correlations between vitamer (HET, HMP, FAMP) concentration and bacterial production indicate vitamer dynamics were linked to bacterioplankton activity. Additionally, positive correlations between dissolved B1, HMP, and FAMP suggest tight cycling of these compounds through synthesis, degradation, release, and/or B1 salvage. All considered, the obtained vitamin data point to planktonic cells as major B1 and vitamer sources (rather than allochthonous sources), even in this shallow, coastal system.

*In-situ* B1 degradation is expected based on culture experiments, abiotic vitamin incubations, and limited vertical profile data [14, 28, 84, 85]. Here, we find strong evidence of ongoing B1 degradation based on positive correlations with temperature (for FAMP) and the observed dynamics for degradation vitamers (HET, FAMP). Two forms of B1 degradation seem to occur in RF during different seasons: 1) temperature or light-driven degradation leading to production of FAMP, a vitamer recently described in the marine environment [5] and 2) generation of HET, canonically thought to occur via hydrolysis of B1 [86], by an unclear pathway. Such patterns have not been previously described but accentuate seasonal B1 cycling processes (generation, uptake, etc.) and *in-situ* degradation (Fig. 2A).

Particulate concentrations of B1 and HMP in RF were lower than those reported from San Pedro Ocean Time Series and a Mediterranean Sea-North Atlantic transect [15, 27]. Change in dissolved B1 or vitamer concentration was not correlated with respective particulate concentration (Supplementary Fig. S3B), indicating that complex transformations are occurring between pools and that dissolved B1 may not predict available particulate B1. Overall, these and additional estimates of B1 and vitamer concentrations per organic carbon will be useful for models of B1 and vitamer flow through aquatic food webs and resulting biomass sustained by such flow [87].

### Bacterial cornerstones that supply vitamin B1

Because B1 production per cell is not well constrained [12, 14] and auxotrophy/prototrophy in eukaryotic plankton is challenging to determine *in-situ* (due to challenges in reconstruction of environmental eukaryotic genomes), a significant unknown remains unanswered—*who are key producers of B1 in-situ?* In RF, biomass estimates of major eukaryotic plankton groups were not correlated with B1 or vitamers except particulate HMP (Supplementary Table S7)—thus, eukaryotic phytoplankton do not appear to be key net sources of B1 (as well as vitamers overall). Also, many common RF taxa were putative auxotrophs based on culture studies (*Mamiellales*, *Chlorophyta*; *Euglenophyta*; *Cryptophyta*) [12], except dinoflagellates and diatoms [8, 12]; Fig. 3A, Supplementary Fig. S6). No *thiC* transcripts classified as eukaryotic were detected, and unclassified *thiC* transcripts were consistently lower than bacterial *thiC* transcripts (Supplementary Fig. S14). High abundances of eukaryotic *thiC* transcripts have been reported from Diatom cultures [21], possibly the eukaryotic contribution is underestimated here by the methods selected for gene calling and mapping of metatranscriptomic reads to metagenomic assemblies. Nonetheless, there was no significant relationship between eukaryotic biomass and vitamin concentrations and a prevalence of putative auxotrophic phytoplankton taxa in RF.

Within prokaryotes, abundant B1 synthesizers were picocyanobacteria and BACL14 (*Methylophilaceaea*, *Burkholderiales*) and to a lesser extent *Verrucomicrobiales* (Fig. 6B). Together, these few bacterial populations accounted regularly for more than 75% of *thiC* transcripts. These groups are widespread and show little variation in B1-related genotype—strongly pointing to them as key B1 sources in coastal waters and beyond [11, 22, 88–90].

*Cyanobacteria* are key primary producers in marine and brackish systems [91–94] and “rhythm setters” for heterotrophs reliant on their organic carbon production [95]. Our *in situ* data extend their importance to include increasing B1/vitamer concentrations in dissolved and particulate pools (based on correlations, Supplementary Table S7), which has repercussions upon auxotroph survival and B1 trophic transfer. The mechanisms of vitamin provision may involve interactions [39, 96] or cell mortality [39, 97]. Nonetheless, vitamin measurements and correlations point to picocyanobacteria as key sources in RF, and this fits with the observation of increased B1 and/or vitamers [36], HMP, and pseudocobalamin [13, 98] availability in cyanobacterial cultures. When cyanobacteria are rare, heterotrophic BACL14 (*Burkholderiales*) and *Verrucomicrobiales* serve as prominent B1 sources based on *thiC* transcripts. These heterotrophs use different organic carbon sources and globally occur across freshwater and marine systems [99–102]. Speculatively the variety of lifestyles *de novo* B1 synthesizers possess helps maintain ecosystem B1 availability across seasons and potentially in microenvironments (e.g. on particles).

### Key B1-related physiology in bacteria based on genomic and transcriptomic profiles

Active use of exogenous B1 (or phosphorylated B1) was evident in RF based on B1 transporter transcripts (*thiB*, *thiT*, Fig. 4D). While *thiB* was present in diverse taxonomic groups (*Actinobacteriota*, *Alphaproteobacteria*, *Gammaproteobacteria*), as previously observed [11], *thiT* has not been described in marine prokaryotes and was specific to *Firmicutes* (Supplementary Fig. S11) [103]—possibly highlighting a unique avenue to obtain B1. Further, we could link dynamics of vitamer concentrations and the prevalence of (putative) vitamer transporter transcripts (*thiV*, *thiY*), which indicates that salvage of B1 from pyrimidine vitamers was another strategy to meet cellular B1 demands, as seen by the *Pelagibacterales* clusters (Fig. 5B).

Certain microbial populations encoded salvage enzymes (*tenA*, *thiM*) indicating vitamer transformation processes take place in the coastal microbial community and population-specific vitamer usage. Transcription of *thiM* by Cluster 56, *Puniceispirillales* (formerly SAR116 clade) links B1 cycling to the sulfur cycle of surface waters [104]. Overall, *tenA* was predominantly detected in pyrimidine auxotrophs highlighting the importance of vitamer salvage to fulfill B1 requirements (e.g. *Pelagibacterales IMCC9063*). Hence, select and abundant microbial populations maintain the capacity to use degradation products, despite apparent availability of B1, emphasizing the evolutionary advantage of using degradation products.

**Figure 7:**
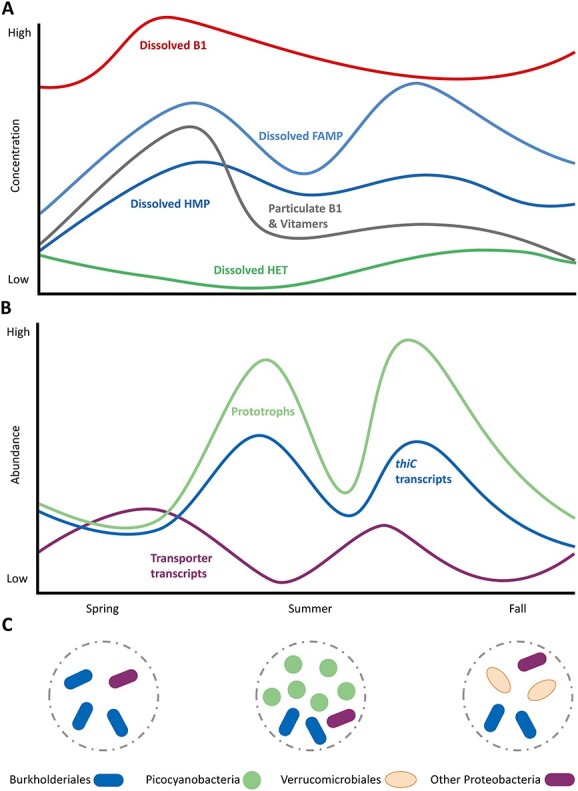
Proposed seasonal dynamics of B1 and vitamer concentrations (A) in connection to relative abundance of prototrophic bacterioplankton and transcription of *thiC*, indicative for *de novo* synthesis, and transporter transcripts (B); seasonal main prototrophs of the microbial community are indicated in the circles (C).

Jointly synthesizing our *in-situ* vitamin measurements and genomics data from RF yields a new holistic view of B1 cycling in coastal waters—one where seasonal dynamics of B1/vitamers are distinct and prototrophic (picocyanobacteria, BACL14, *Verrucomicrobiales*) and auxotrophic (*Pelagibacteriales*, Actinobacteria) taxa are key in cycling (Fig. 7). The improved view of B1 and vitamers dynamics, and key producers serves as a “model” for comparison to other regions of the ocean, especially open ocean, as well as a guide for future mechanistic studies to explain *in-situ* phenomena, e.g. stronger change in vitamer concentration. Key B1 producers identified here, especially ubiquitous picocyanobacteria, are now clear targets for studies that assess rates and types of vitamin/vitamer provision. A combined understanding of *in-situ* dynamics and mechanistic drivers will yield greatest predictive power—here we provide valuable information on the prior, specifically within coastal brackish water.

## Supplementary Material

2-SI_Roskilde_manuscript_20231215_combined_ycad016

## Data Availability

Ribosomal RNA gene sequences, metagenomic, and metatranscriptomic sequence data are deposited at the EMBL databases under study accession number PRJEB59221. The bioinformatic pipeline for the metagenomic processing is available at https://github.com/EnvGen/B1-Ocean, while the pipeline for mapping and normalization of metatranscriptomic reads can be found at https://github.com/johnne/map_metaT. Supplementary information and Supplementary Fig. S11 are available on the journal’s website. Source data for main figures, high-resolution Supplementary Figs S11 and S12, and Supplementary Tables as excel files can be found on figshare DOI: 10.6084/m9.figshare.23634429 and 10.6084/m9.figshare.23634465, respectively.
