# Supplementary material for: New chemical and microbial perspectives on vitamin B1 and vitamer dynamics of a coastal system
Source: ISME Commun. 2024 Jan 10;4(1):ycad016. doi: 10.1093/ismeco/ycad016 (PMC10881298; doi:10.1093/ismeco/ycad016)
Supplement: 2-SI_Roskilde_manuscript_20231215_combined_ycad016 [file 2-si_roskilde_manuscript_20231215_combined_ycad016.pdf]

Supplementary Information

New chemical and microbial perspectives on vitamin B1 and vitamer dynamics of a coastal system

Meriel J. Bittner[1], Catherine C. Bannon[2], Elden Rowland[2], John Sundh[3], Erin M. Bertrand[2], Anders F. Andersson[4], Ryan W. Paerl[5], Lasse Riemann[1]

[1]Marine Biological Section, Department of Biology, University of Copenhagen, Helsingør, Denmark

[2]Department of Biology, Dalhousie University, Halifax, Nova Scotia, Canada

[3]Department of Biochemistry and Biophysics, National Bioinformatic Infrastructure Sweden, Science for Life Laboratory, Stockholm University, Solna, Sweden

[4]Department of Gene Technology, Science for Life Laboratory, School of Engineering Sciences in Chemistry, Biotechnology and Health, KTH Royal Institute of Technology, Stockholm, Sweden

[5]Department of Marine, Earth and Atmospheric Sciences, North Carolina State University, Raleigh, North Carolina, USA

Contents of this file

Supplementary Methods 1 to 5

Supplementary Figures S1 to S10, S12-S13

Supplementary Tables S1-S9

References for Supplementary Information

Additional Supplementary files (uploaded separately)

Supplementary Figure S11

Supplementary Materials (DOI: 10.6084/m9.figshare.23634465) and Source Data (DOI: 10.6084/m9.figshare.23634429) can also be found on figshare.

Supplementary Methods

1. Vitamin B_1 and precursor amendment experiment

Two nutrient amendment experiments (23 June and 18 August 2020) were carried out to test for potential B1 or vitamer (HMP+HET) limitation compared to a control treatment. In the B1 treatment incubation, bottles were amended with 1 nM of B1 (Sigma Aldrich, ≥99% purity). In the vitamer treatment, HET (Sigma Aldrich, ≥95% purity) and HMP (Enamine Ltd, >95% purity) were each added at 1 nM final concentration. These final concentrations have been previously used in an amendment experiments in the Baltic Sea [1] and were considered suitable to test for B1/vitamer limitation in the productive Roskilde fjord in light of other coastal dissolved B1 concentrations with occasionally up to a few hundred pM [2]. Bottles were incubated for 40 or 48 h in the dark at near in situ temperature and inverted approximately every 6 h. Bacterial production was measured at three or four time points (Supplementary Methods 3). At the end of the incubation experiment from 18 August, water samples (200 ml) from each replicate bottle per treatment was pooled and filtered onto one a 0.22 µm GVWP filter (Millipore) used for metagenomics to increase read coverage for bins originating from taxa with low abundances (Supplementary Table S3).

2. Particulate organic carbon

For particulate organic carbon (POC) analysis, duplicate samples of 2 L seawater were filtered onto pre-combusted GF/F filters (Whatman). Filters were washed with 10 mL MilliQ to remove salts and stored at -20°C until processing. Filters were then dried (1 h, 55°C), acidified with 0.2 M HCl to remove particulate inorganic carbon, and dried again (20 h, 55°C). A quarter of each filter was encapsulated and analyzed with a Shimadzu Total Organic Carbon / Nitrogen Analyzer TOC-5000A. Two blank filters were prepared the same way but with 1 L MilliQ filtered. The average of the blanks was subtracted from each measurement.

3. Heterotrophic bacterial production

Heterotrophic bacterial production was estimated by leucine incorporation (L-[4,5-3H]-Leucine, NET1166001MC, Perkin Elmer, specific radioactivity 180 Ci $mmol^{-1}$) [3]. A 1:10 or 1:50 hot:total leucine solution (5 µM final concentration) was freshly prepared before each

sampling and triplicate samples were amended with 68 µl of radioactive leucine solution (final concentration 200 nM leucine) in 2 mL microcentrifugation tubes.

Two samples killed immediately after leucine addition with 100% TCA (5% final) served as blanks. Incubation at *in situ* temperature in the dark was terminated after 1 h with 100% TCA (5% final). Samples were stored at 4°C until further processing and analysis. After reaching room temperature, samples were centrifuged for 10 min at 17,000 g, supernatants were aspirated, and the cell pellets were washed twice with 5% TCA. Afterwards, cell pellets were resuspended in 1 mL liquid scintillation cocktail (Ultima Gold AB, 6013301, Perkin Elmer) and incubated for ~24 h at room temperature in the dark. Incorporated radioactive leucine was determined by a liquid scintillation analyzer (Tri-Carb 2910 TR, Perkin Elmer). The averages of technical triplicate measurements of the disintegration per minute (DPM) were corrected for the average of the two blanks. For calculating bacterial production the conversion factors of 7.8×10^{16} cells per mol leucine [4] and 20 fg C per cell [5] were applied.

4. Vitamin and vitamer sample collection and analysis

4.1 Sample collection

Before each sampling, filtration units and brown HDPE bottles were cleaned with methanol and MilliQ. Replicate samples of 500 mL were filtered with a low vacuum (< 0.17 mbar) onto 47 mm nylon filters (0.20 µm, GVS North America). Filters for particulate vitamin analysis were folded with sterilized tweezers into cryovials and stored at -80°C. The filtrate of one replicate was used to rinse the bottles for the other replicates. Filtrates for dissolved analysis were stored in brown HDPE bottles at -20°C until further processing.

4.2 Particulate B-Vitamin extraction

The procedure was adapted from [6] and [7]. The sample preparation was conducted in a dark room with a red-light source and samples were kept on ice whenever possible. An internal standard mixture, including stable isotope labeled B1 (10 pm of ^{13}C3 thiamine-HCl, Cambridge Isotope Laboratories, CLM-7667) was added to each filter.

To each sample 0.2 mL each of 100 and 400 µm silica beads (OPS Diagnostics, Lebanon, NJ) along with 1000 µL of ice-cold extraction solvent (40:40:20 acetonitrile:methanol:water) were added. A bead beater (MP Biomedicals) was used to agitate the cells in 3 equally spaced 40 second pulses at 1800 rpm over a 20 min period, with the samples placed on ice between

agitations. The tubes were briefly centrifuged, and the supernatant transferred to a clean 2 mL tube. 300 µL of solvent mixture (40:40:20 acetonitrile:methanol:water) was added to rinse the filter, the tube centrifuged and the solvent added to the primary extract. Two additional rinses of the filter and tube were performed with 300 µL of ice-cold methanol and all extracts pooled. The samples were dried with a Vacufuge (Eppendorf, Mississauga, ON) at room temperature and stored at -80 °C until analysis. The percent recovery for internal standards was >90% as determined from calibration curves generated with authentic standards. For mass-spectrometry analysis, samples were re-suspended in 100 µL of 20 mM ammonium formate, 0.1% formic acid, 2% acetonitrile on ice. Samples were briefly vortexed, centrifuged at 14,800 rpm for 10 min at 4 ºC. Then aliquots were added to conical polypropylene HPLC vials (Phenomenex, Torrance, CA) and diluted four-fold prior to analysis.

4.3 Dissolved B-Vitamin capture and processing

Dissolved samples were concentrated on C18- solid phase extraction (SPE) cartridges. SPE columns (Waters, WAT043345) were conditioned by soaking overnight in HPLC-grade methanol and washed with 25 ml HPLC plus grade water pumped over cartridges at ca. 1 ml min-1 flow rate. All tubing and cartridge adapters were cleaned with methanol and MilliQ. Frozen filtrate bottles were thawed overnight at 16°C before adjusting the pH to 6.5 using 1 M molecular grade HCl. Dissolved samples were spiked with stable-isotope (13C) labelled vitamin B1 (thiamine-(4-methyl-13C-thiazol-5-yl-13C3) hydrochloride, Sigma Aldrich, 731188) to a final concentration of 75 pM. Each replicate was pumped over a SPE column (~1 mL min^{-1}), the column washed with 200 mL HPLC grade water by gravitational flow and purged of water. Cartridges were wrapped in combusted aluminum foil, sealed and stored in individual bags at -20°C until processing.

Columns were thawed at room temperature for 30 min, placed in a vacuum manifold (Waters) and washed with 100 mL HPLC grade water before gently purging residual water and eluting with 35 mL methanol. All vacuum manifold steps were performed with less than 5 in Hg vacuum, resulting in a ca. 5 mL min-1 flow rate. Solvent was removed using a roto evaporator (Centrivap, Labconco) over 12 to 24 hr. For some samples, a white pellet, which may have been leaked SPE C18 resin, was observed after drying. These samples were resuspended in 1 mL of methanol and filtered through Pierce 30 µm pore size spin filters

(Thermo Scientific) by gravity and the eluant again dried by roto evaporator. The samples were resuspended in 200 µl HPLC buffer A and 2-fold dilutions were performed for analysis.

4.4 Mass spectrometry analysis

HPLC-MS was used to quantify selected metabolites using a Dionex Ultimate-3000 LC system. Chromatographic separation was achieved using a T3 C18 column (100Å, 1.8 µm, 300 µm X 150 mm, Waters). The HPLC was coupled to the electrospray ionization source of a TSQ Quantiva triple-stage quadrupole mass spectrometer (ThermoFisher) operated in SRM mode, with the following settings: Q1 and Q3 resolution 0.7 (FWHM), 6 ms dwell time, CID Gas 2.5 mTorr, spray voltage in 3500 positive ion mode, sheath gas 6, auxiliary gas 2, ion transfer tube temperature 325 ºC, vaporizer temperature 100 ºC. Duplicate 5 µL injections were performed onto a 300 µm x 150 mm column (nanoEase, M/Z HSS T3 Column, 1.8 µm, 100 Å) with a 300 µm x 50 mm guard column in front (nanoEase M/Z HSS T3 Trap Column, 5 µm, 100 Å), held at 45 °C and subject to an HPLC gradient of 4 – 99% B over 8 min (buffer A, 20 mM ammonium formate, 0.1% formic acid; buffer B, 0.1% formic acid in acetonitrile) at 8 µl per min. The total run time including washing and equilibration was 12 min. The transition list (parent and fragment mass values for compounds targeted) can be found in Supplementary Table 1.

4.4.1 Vitamin and Vitamer quantification

A pooled quality control (QC) sample was prepared by mixing equal volumes of each sample within each sample group (particulate and dissolved). The QC sample was injected after every 5 samples throughout the mass spectrometry analysis to monitor instrument response and compound degradation. Furthermore, the QC injections were used to determine best matched internal standards for normalization purposes to reduce the matrix effect and variability introduced during sample processing.

The method of standard addition was used to quantify compounds of interest. Calibration curves were used to determine the concentration of each target already in the pooled QC sample. Using authentic standards, calibration curves were prepared for each matrix grouping. Triplicate injections were performed at 0, 1, 5, 10 and 50 fmol on column HET (Sigma) and at 0, 5, 25, 50, and 250 fmol on column for B1 (ThermoFisher), TMP (Sigma), HMP (Enamine Ltd. Kiev, Ukraine), cHET (Finetech Industry Limited, Wuhan, China), FAMP and AmMP (both Toronto Research Chemicals, Toronto, Canada).

4.4.2 Data analysis

Data analysis was adapted from [4] and [5]. Briefly, raw files generated with Xcalibur software (ThermoFisher) were uploaded into Skyline Daily (University Washington) and the transitions with the best signal to noise and lowest interference were selected for quantification purposes. Peak areas were exported and processed in MS Excel or R. Metabolites were normalized to their corresponding heavy internal standards. which reduced variability introduced by matrix effects and sample preparation error. Limits of quantitation (LOQ) and limits of detection (LOD) were calculated as 10x and 3x, respectively, the variation in the inter-sample blanks for all compounds. For calculation of limit of detection, the standard variation in sample QC was used rather than the inter-sample blanks. Additionally, chromatograms for samples with compound concentrations that fell between LOD and LOQ were visually inspected and analyzed in a batch per batch method based on the following criteria, as modified from Boysen et al. (2018): A compound was deemed quantifiable in samples that fell below the calculated LOQ if, a) the peak was at the same retention time (±0.2 min) as the authentic standard, b) two daughter fragments were present with co-occurring peaks, c) daughter fragments were present in same order of intensity as authentic standard and d) the integrated peak area was at least 2 times greater than the average peak found in the blanks in the appropriate retention time window.

4.5 Discussion on method for vitamin and vitamer measurements

AmMP and cHET could not be detected or quantified, likely due to a combination of low environmental concentrations and low signal to noise ratios for these compounds. In particular, the LOD and LOQ for AmMP are high relative to the other vitamers (Supplementary Table S1), and thus more difficult to detect. Previously these vitamers have only been measured in the dissolved phase in surface waters in the North Atlantic where they were in the low picomolar ranges [8]. While we were able to reliably measure B1, we could not reliably quantify the phosphorylated forms of B1 (TMP, TDP), which contribute to the B1 pool [9, 10].

We used minimum three biological replicates to determine B1 and vitamer concentrations. This is often not possible due to limitations in sample volume during field campaigns [8]. The variation between biological replicates most likely originates from variability in the preconcentration step [11], which we try to account for by applying a B1 recovery value for each biological sample. The remaining observed variability in replicate

dissolved B1 samples could indicate small scale differences and gradients in the aquatic environment [12]. Estimates of dissolved B1 recovery vary [9, 11, 13], likely induced by sample processing, detection method, internal standard preparation, recovery calculation, and calibration curves. We hypothesize that matrix effects during our preconcentration step caused the overall low recovery of dissolved B1, as found earlier and is common with quantification of dissolved marine metabolite [11, 14, 15].

5. Sequence and data analysis

5.1 Analysis of 16S and 18S rRNA gene amplicons

The 16S and 18S rRNA gene reads were processed according to the workflow by Mike Lee (https://astrobiomike.github.io/amplicon/16S_and_18S_mixed – evaluating-the-outcome) as the primer set has been shown to adequately capture 16S and 18S rRNA gene diversity [16, 17]. Primers were trimmed with cutadapt (v3.4) [18]. To identify 18S rRNA gene reads, samples were run with Magic-BLAST (v1.5.0) [19] against the 18S rRNA gene sequences of the PR2 database (v4.14.0) [20]. Accordingly, reads of each sample were split into 16S and 18S rRNA gene reads. These were filtered in parallel, trimmed where read quality dropped below 30 (16S: F 250, R 190; 18S: F230, R 190), dereplicated, read pairs were merged (for 16S: minOverlap = 60) and chimeras removed in dada2 (v1.22.0, https://benjjneb.github.io/dada2/index.html).

Further processing in phyloseq (v1.38.0) [21] included removal of sequences from unclassified phyla (including mitochondria sequences), standardizing abundances to median sequencing depth and removal of singletons. This resulted in 3,641 and 883 ASVs for 16S and 18S rRNA gene libraries, respectively.

5.2 Metagenomes and metatranscriptomes

Metagenomic and metatranscriptomic data was processed with a snakemake pipeline (https://github.com/EnvGen/B1-Ocean) based on the nbis-meta workflow (https://github.com/NBISweden/nbis-meta). In brief, metagenomic samples were quality checked with FastQC (v0.11.9 https://www.bioinformatics.babraham.ac.uk/projects/fastqc/) and Illumina TrueSeq Universal adapters were trimmed by cutadapt (v2.6) [18]. Single assemblies were generated for each of the 17 samples and a set of three co-assemblies were

performed with MEGAHIT (v1.2.9) with settings '–min-contig-len 300 –prune-level 3' in addition to the default [22] (Supplementary Table S3). Genes were predicted with Prodigal (v2.6.3) [23] using the '-p meta' mode, and functionally annotated with infernal (v1.1.2) for rRNA identification [24], eggnog-mapper (v2.1.2) with v5.0 of the EGGNOG database to infer KEGG orthologs, pathways and modules [25].

Further, hmmsearch (v.3.3.2, e-value cutoff 0.001) (http://hmmer.org) was run to identify non-overlapping proteins based on the Pfam database (v31.0) [26] plus additional HMM profiles for B1-related biosynthesis, salvage and transporter proteins obtained from (TIGRFAM v15.0, https://doi.org/10.6084/m9.figshare.5928151.v1) [1, 27]. Protein annotations related to B1 were further verified and if necessary, filtered by a custom cutoff (see Supplementary Methods 5.3). Assembled contigs were taxonomically annotated with contigtax (UniRef100, v2021_04, https://github.com/NBISweden/contigtax) and sourmash (v4.2.2, gtdb-rs202 database) [28]. Additionally, thiamin diphosphate riboswitches (THI-box) were identified on contigs with the TDP riboswitch model (TPP, RF00059) with infernal [29, 30].

A subset of assemblies was binned with MetaBAT2 (v2.14) [31] and CONCOCT (v1.1.0) [32] with minimum contig lengths of 1500 and 2500 bp each. After evaluating the four binning approaches in terms of bin completion, contamination and number of bins across bin quality groups, we continued with MetaBAT2 and 1500 bp minimum contig length. Bin quality was assessed with CheckM (v1.1.2) [33] and bins were phylogenetically classified with GTDB-Tk (v2.1.0) [34]. Average nucleotide identity of binned genomes was calculated with fastANI (v1.3) [35]. Bins considered medium- or higher-quality draft metagenome-assembled genomes (MAGs; ≥50% completion, <10% contamination) [36] were clustered at 95% identity [37], and a list of reference genomes (Supplementary Table S6) was used to aid clustering. For gene abundances on the assembly level, values were normalized to selected single-copy marker gene abundances (Supplementary Table S7).

Metatranscriptomic samples were processed with FastQC and trimmomatic (v0.39) for quality and adapter trimming. To remove rRNA reads, sortMeRNA (v2.1b) [38] was run with all rRNA databases before continuing with mapping reads to the metagenomic assemblies with bowtie2 (v2.4.5) [39]. Annotations for the metatranscriptomic reads were obtained from corresponding mapped metagenomic reads. Metatranscriptomic profiles on assembly level were normalized according to [40]. Gene abundances on assembly level were

divided by the median abundance of selected single-copy marker genes. For KEGG orthologs (KO), 10 Kos profiles were used for normalization and 21 Pfam models for normalizing the pfam-hmmsearch counts (Supplementary Table S7).

5.3 Protein annotations

Protein annotations from hmmsearch were checked for a subset of three samples (3 March, 8 July, 4 November 2020). Five criteria were evaluated to verify the hmmsearch protein annotations: (i) amino acid residues interacting with the B1 or vitamer molecule (if described), (ii) protein phylogeny, (iii) additional protein annotation, (iv) TDP riboswitch upstream of the ORF (if applicable), and (v) other B1 related genes in proximity of the ORF. For this, amino acid sequences were aligned (MUSCLE, 3.8.425) and manually inspected in geneious (2020.2.5). Further, protein phylogenies were constructed (RaxML, v8) and, in some cases, reference sequences were included in the alignments to help with verification. Next, amino acid sequences were run with blastp [41] against the RefSeq-protein database (September 2022) [42]. In case of no close hits, sequences were additionally annotated with UniProtKB [43] or the NCBI protein database. Information from all five criteria was used to determine if annotations were correct.

Based on this analysis, we adjusted the protein cutoffs for six proteins to remove false positive hits while keeping true positives, representative of environmental protein diversity (Supplementary Table S5). The protein cutoff for ThiG and ThiE was set higher than the trusted cutoff listed in the models. Most false positive hits for ThiG were annotated as imidazole glycerol phosphate synthase or pyridoxine synthase when run against RefSeq. Gene counts for *thiC*, *thiG* and *thiE* were compared to gene counts obtained from enzyme annotation. The cutoff for proteins annotated as ThiM, ThiY, ThiV and ThiB were set to lower values than the trusted cutoff (Supplementary Table S5). Environmental diversity of the proteins might be higher than captured so far with the proteins used in the alignments for making current hmm protein models. False positive hits for ThiV get annotated as sodium-proline symporter and false positive hits for ThiB are mostly related to iron uptake.

Supplementary Figures

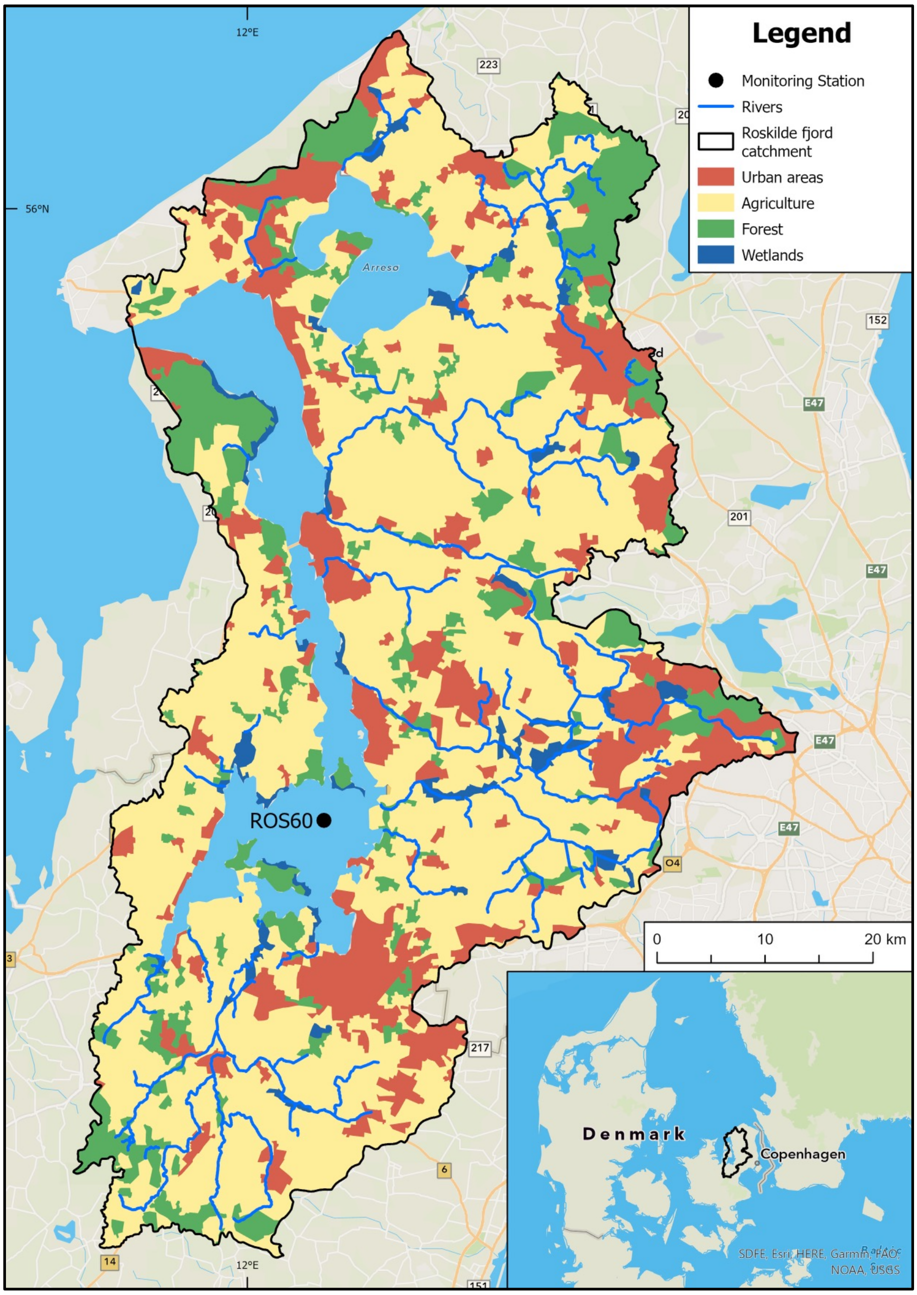

Figure S1: Map depicting the sampling location, monitoring station ROS60, and the catchment area of Roskilde fjord (black contour). Colors indicate land cover in the catchment [44].

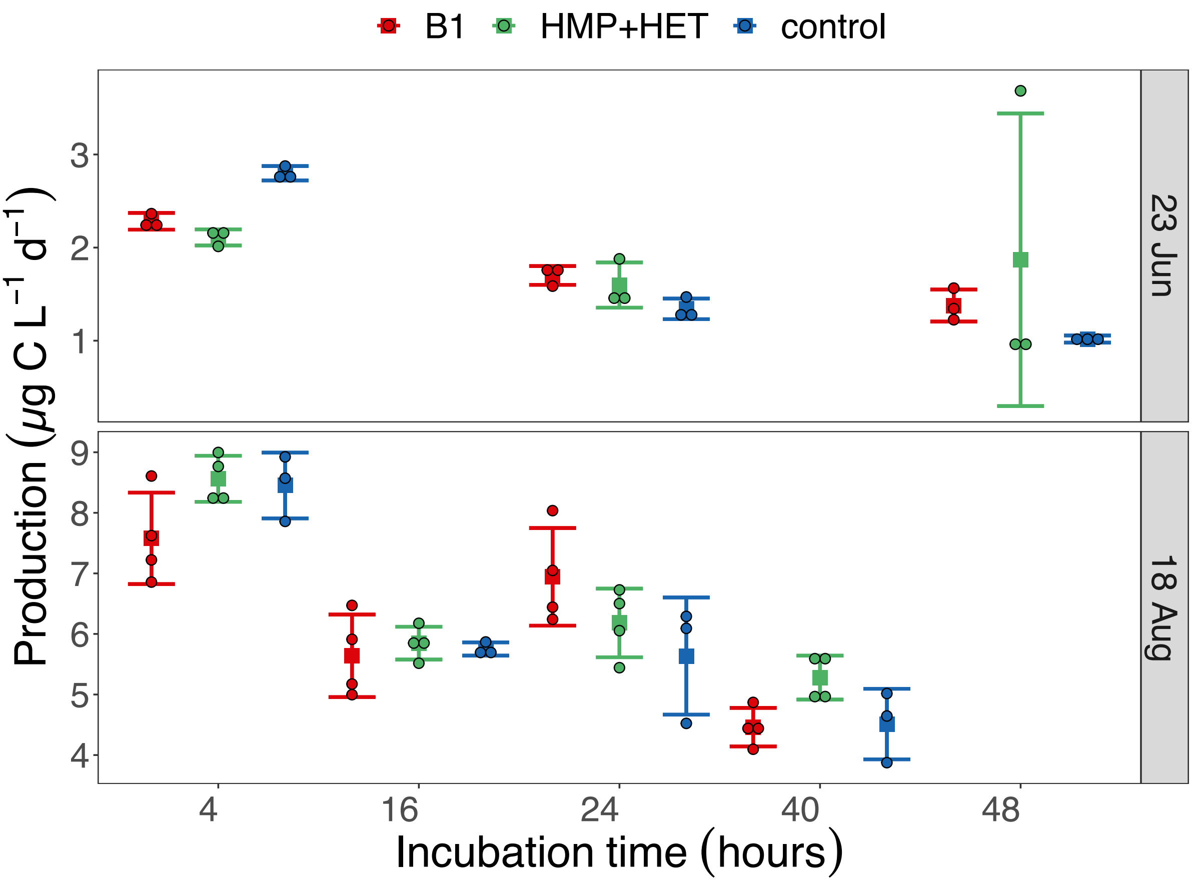

Figure S2: Bacterial production for B1 and vitamer (HMP+HET) amendment experiments and control incubations. For details see Supplementary Methods 1.

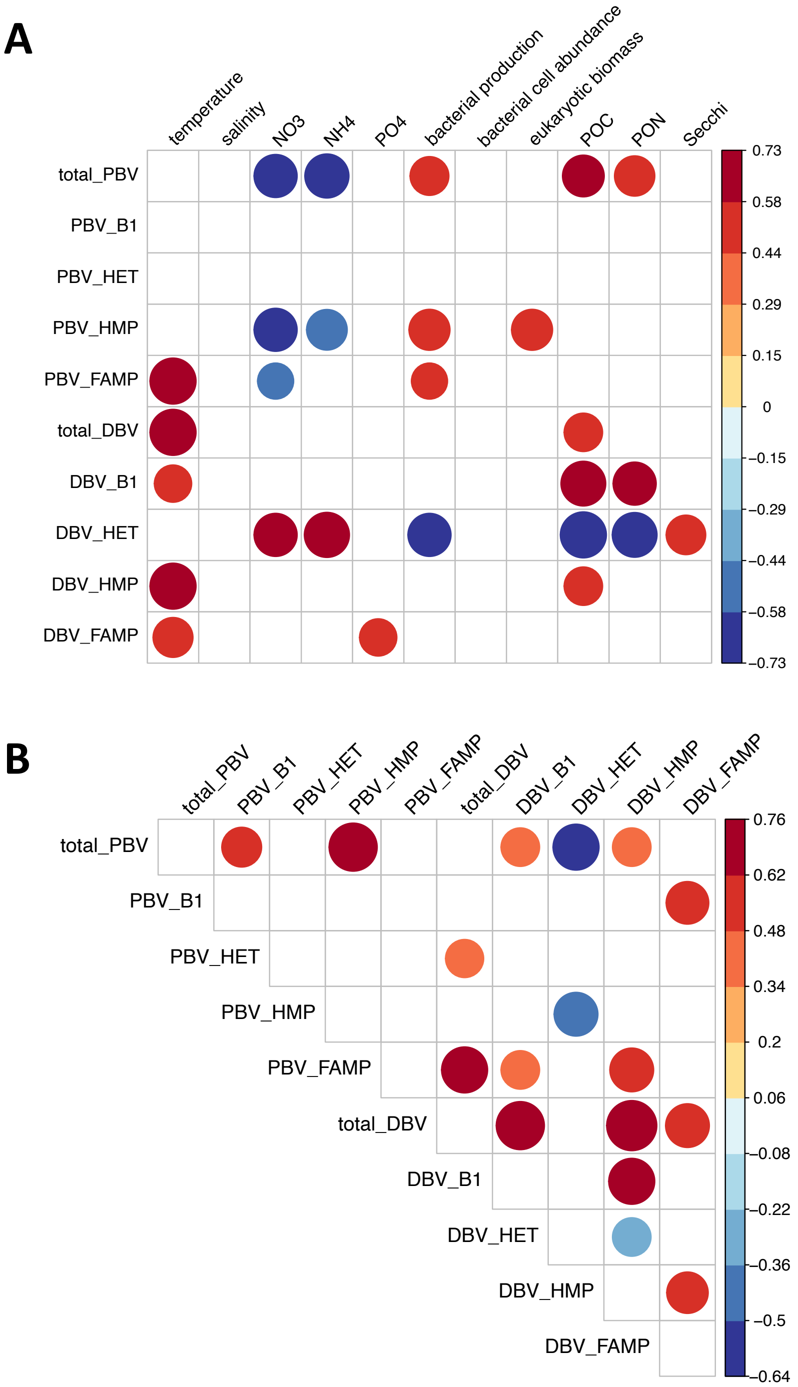

Figure S3: Kendall correlation matrix for vitamin/vitamer concentrations with environmental parameters (**A**) and between vitamin/vitamers (**B**). Colored circles indicate significant correlations ($p < 0.05$) with correlation coefficient tau, white boxes indicate no significant correlation.

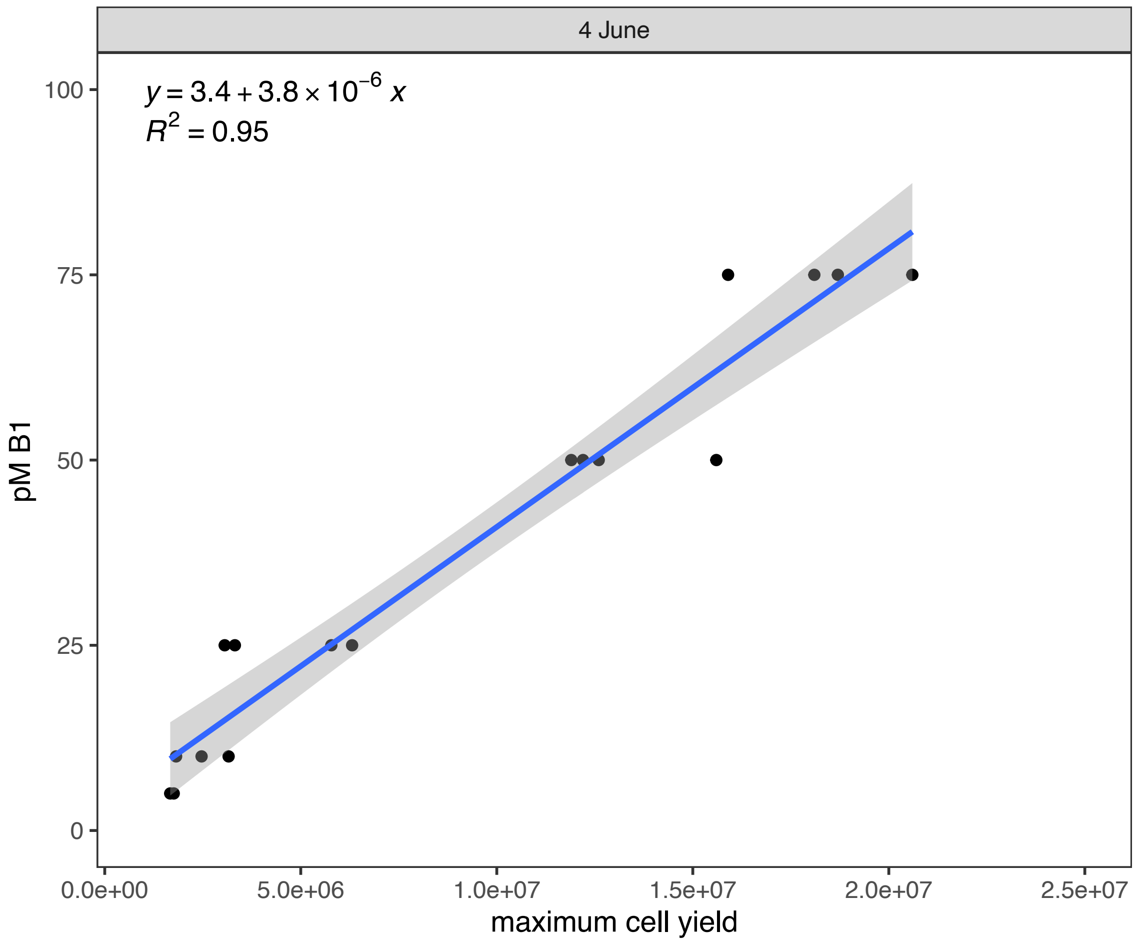

Figure S4: Standard curve for *V. anguillarium* PF430-3 Δ*thiE* in water from Roskilde fjord from 4 June 2020. Standards were run in four replicates and average maximum cell yields from no B1 addition (0 pM) were subtracted from maximum yields with added B1 (5, 10, 25, 50, 75 pM). Maximum cell yields are provided in Supplementary Table S8. No growth was observed in negative controls (growth in B1-deplete medium).

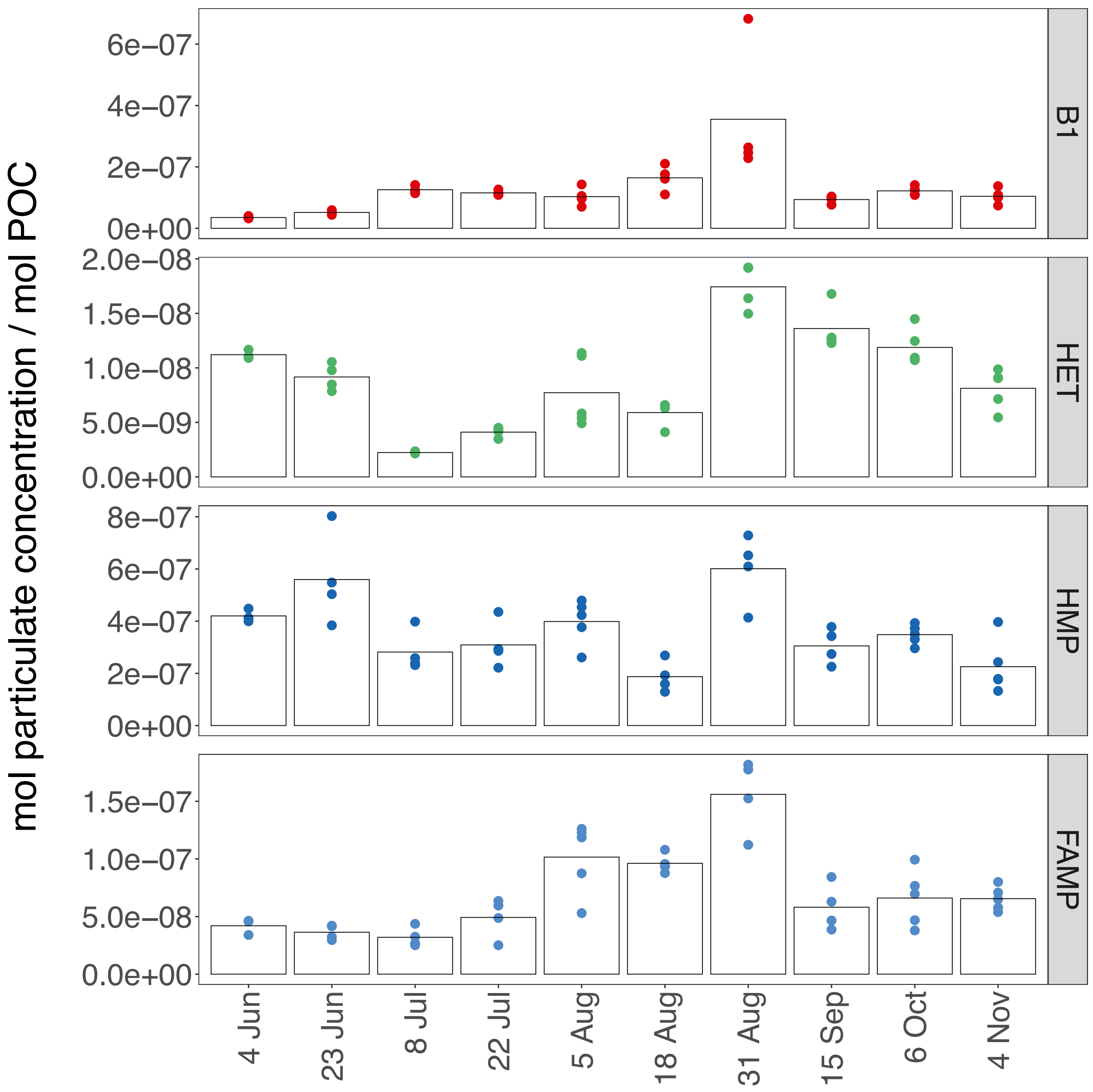

Figure S5: Concentrations of particulate B1 and vitamers normalized to particulate organic carbon (POC). POC data is shown in Figure 1D and available in the source data.

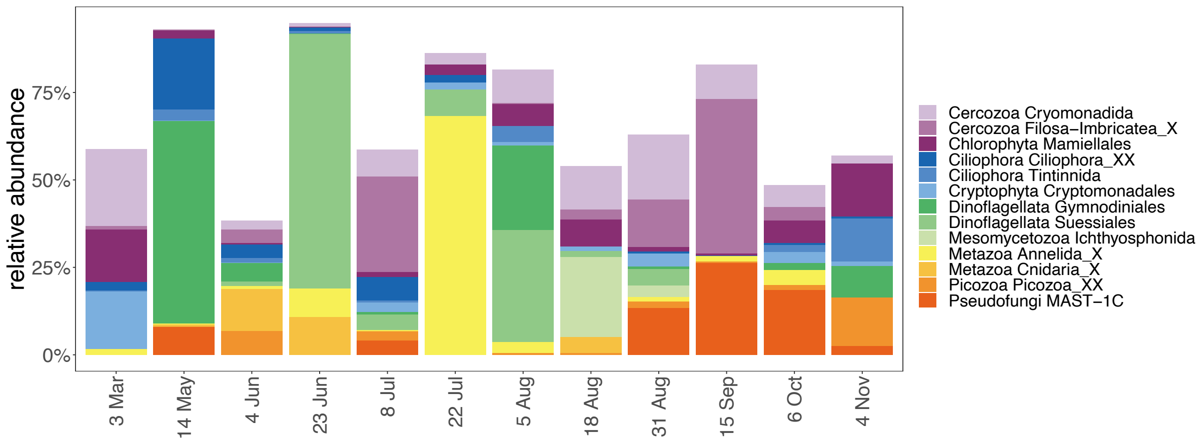

Figure S6: Relative abundances of the top 14 dominant eukaryotic orders based on 18S rRNA gene amplicons obtained from DNA of the size fraction 0.22-90 µm.

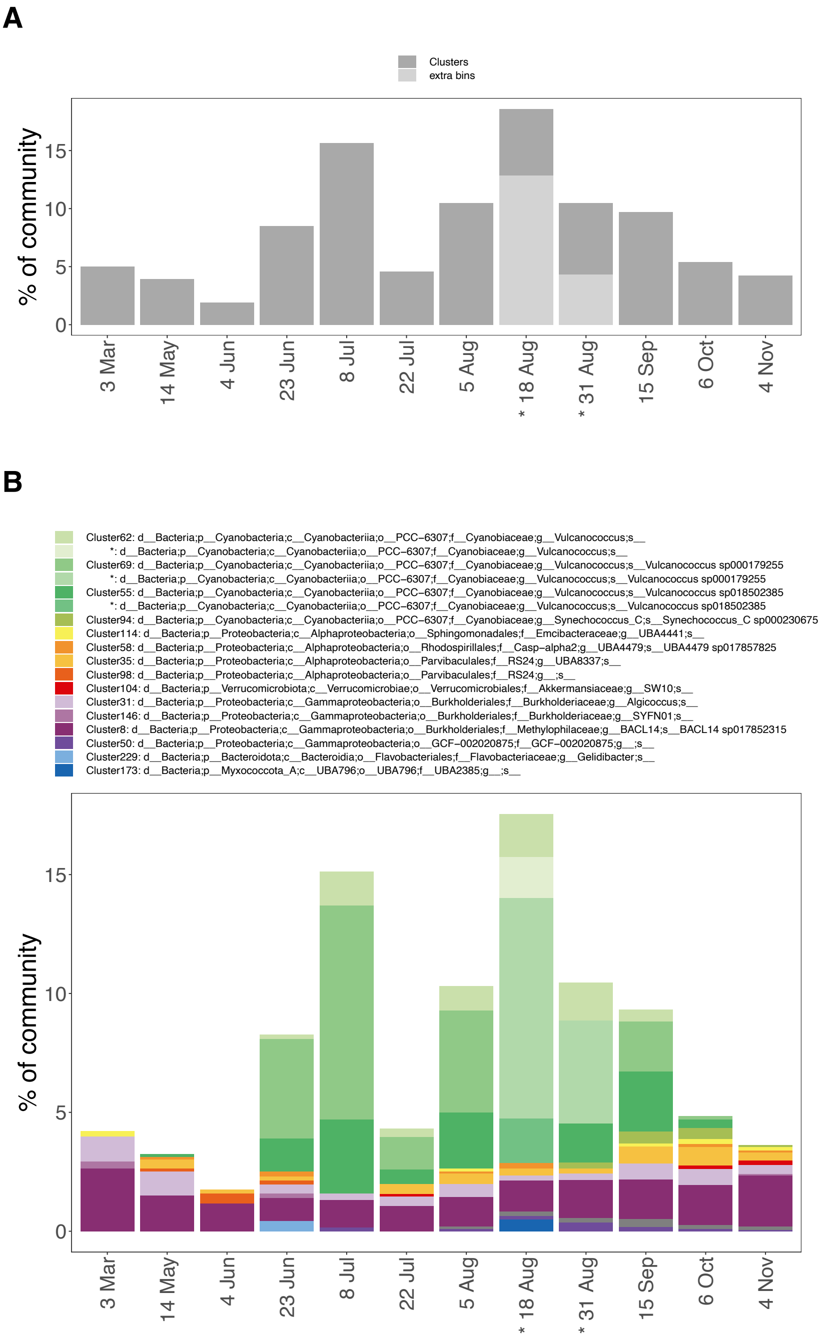

Figure S7: Relative abundance estimate of the most abundant prototrophic clusters and some additional prototrophic bins (A) and their taxonomy (B). Relative community proportion calculated by CheckM, relative to the number of reads mapped to the assembled contigs, adjusted for the size of the bin and reads assigned to bins. Asterisks indicate the three additional bins that were included for 18 and 31 August to get a more accurate abundance estimate for bacterial prototrophs. These three additional bins were not clustered due to a contamination estimate >10%, which is presumably a result of high strain diversity observed in picocyanobacterial genomes at those sampling dates.

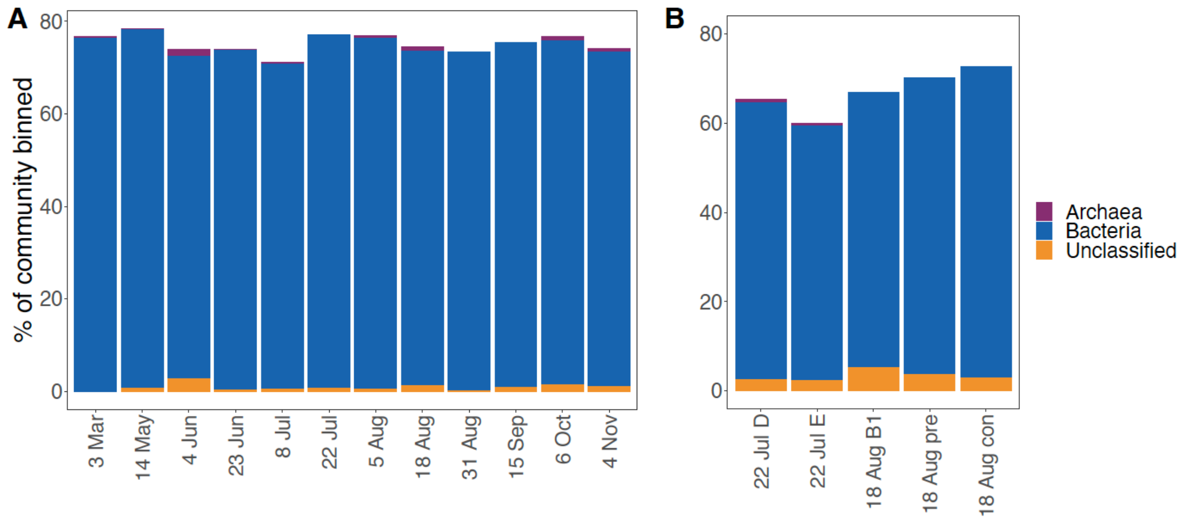

Figure S8: Percentage of reads mapped to bins for single assemblies calculated by CheckM. Main 12 samples (**A**) and 5 additional samples (**B**) shown (Supplementary Table S4).

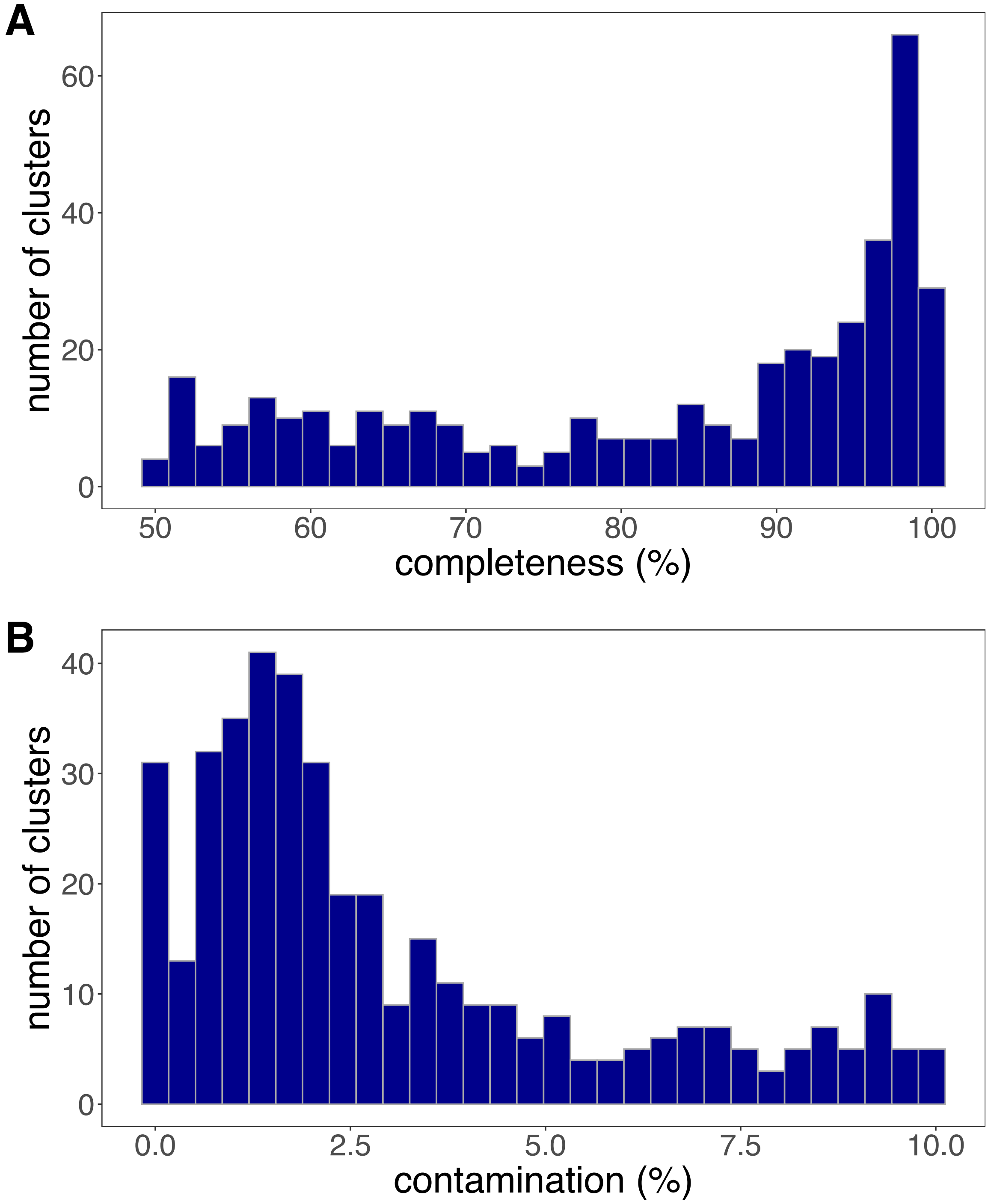

Figure S9: Histograms showing the estimated genome completeness (**A**) and contamination (**B**) of the generated 405 clusters. The bin with the maximum completeness and maximum contamination of each cluster was used for this. Values were estimated by CheckM.

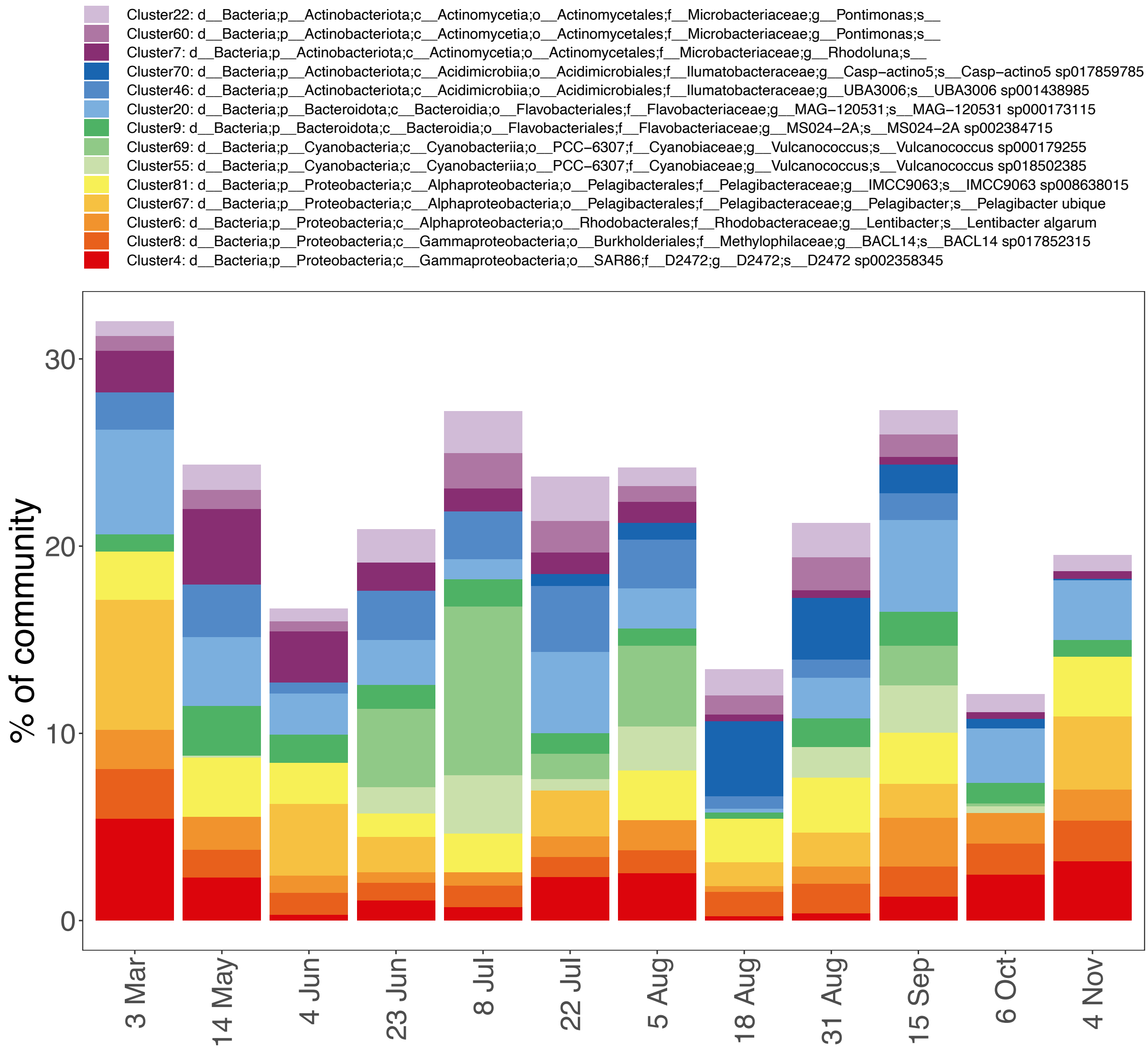

Figure S10: Relative proportions of the top 14 clusters within the microbial community across timepoints. Values were calculated by CheckM, relative to the number of reads mapped to the assembled contigs, adjusted for the size of the bin and reads assigned to bins.

Uploaded separately on journal's website or

can be found on figshare (DOI: 10.6084/m9.figshare.23634465)

Figure S11: B1-related genotypes of 195 clusters with MAGs of high-completion. Heatmap shows presence (dark boxes) or absence (gray boxes) of proteins across clusters. The gradient of the dark boxes indicates the percentage of MAGs containing the protein. Number of MAGs and maximum estimated completion of the MAGs are given on the left side. Taxonomy is shown for each cluster on phylum, order and genus level.

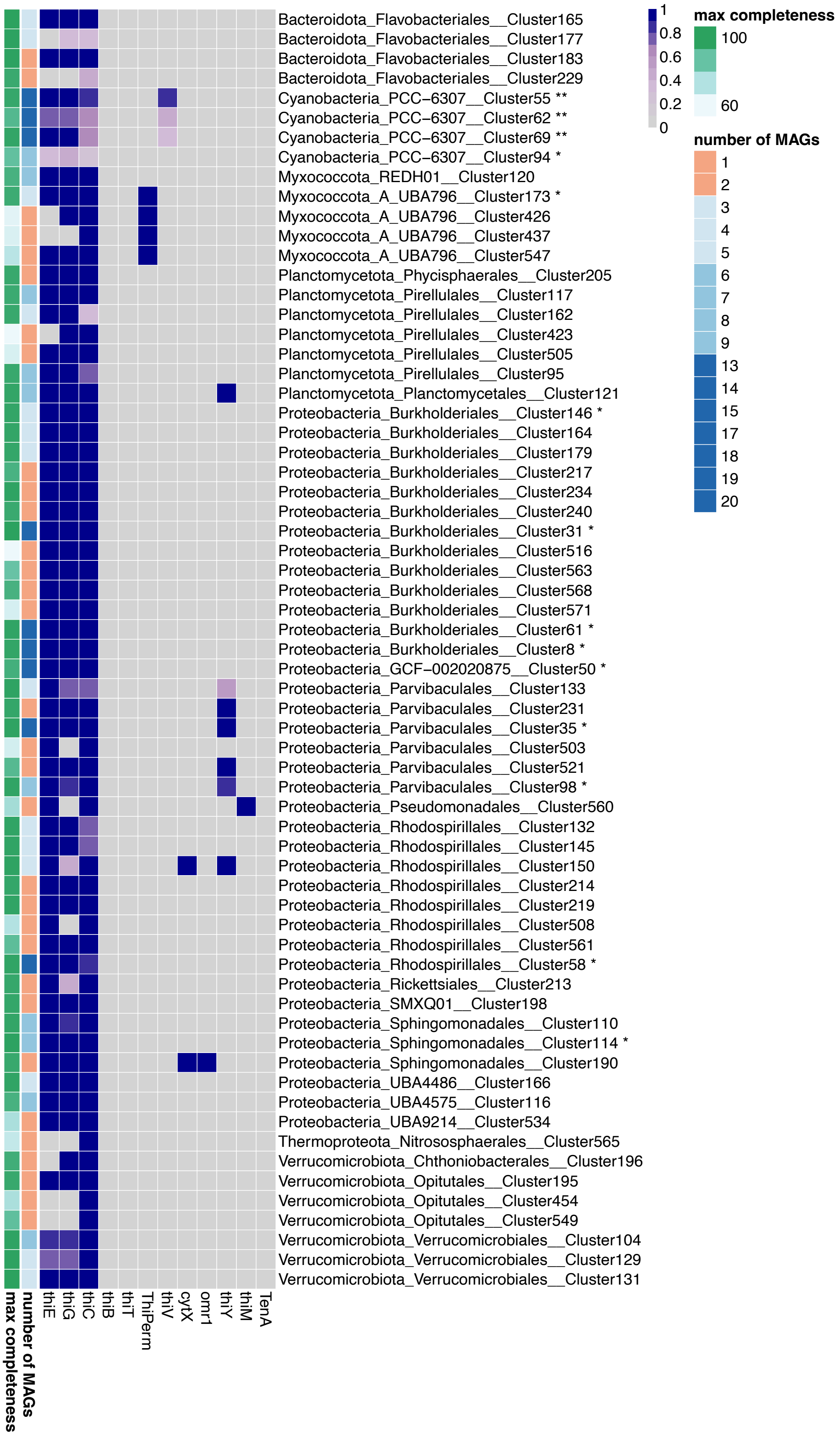
Bacteroidota_Flavobacteriales__Cluster165
Bacteroidota_Flavobacteriales__Cluster177
Bacteroidota_Flavobacteriales__Cluster183
Bacteroidota_Flavobacteriales__Cluster229
Cyanobacteria_PCC−6307__Cluster55 **
Cyanobacteria_PCC−6307__Cluster62 **
Cyanobacteria_PCC−6307__Cluster69 **
Cyanobacteria_PCC−6307__Cluster94 *
Myxococcota_REDH01__Cluster120
Myxococcota_A_UBA796__Cluster173 *
Myxococcota_A_UBA796__Cluster426
Myxococcota_A_UBA796__Cluster437
Myxococcota_A_UBA796__Cluster547
Planctomycetota_Phycisphaerales__Cluster205
Planctomycetota_Pirellulales__Cluster117
Planctomycetota_Pirellulales__Cluster162
Planctomycetota_Pirellulales__Cluster423
Planctomycetota_Pirellulales__Cluster505
Planctomycetota_Pirellulales__Cluster95
Planctomycetota_Planctomycetales__Cluster121
Proteobacteria_Burkholderiales__Cluster146 *
Proteobacteria_Burkholderiales__Cluster164
Proteobacteria_Burkholderiales__Cluster179
Proteobacteria_Burkholderiales__Cluster217
Proteobacteria_Burkholderiales__Cluster234
Proteobacteria_Burkholderiales__Cluster240
Proteobacteria_Burkholderiales__Cluster31 *
Proteobacteria_Burkholderiales__Cluster516
Proteobacteria_Burkholderiales__Cluster563
Proteobacteria_Burkholderiales__Cluster568
Proteobacteria_Burkholderiales__Cluster571
Proteobacteria_Burkholderiales__Cluster61 *
Proteobacteria_Burkholderiales__Cluster8 *
Proteobacteria_GCF−002020875__Cluster50 *
Proteobacteria_Parvibaculales__Cluster133
Proteobacteria_Parvibaculales__Cluster231
Proteobacteria_Parvibaculales__Cluster35 *
Proteobacteria_Parvibaculales__Cluster503
Proteobacteria_Parvibaculales__Cluster521
Proteobacteria_Parvibaculales__Cluster98 *
Proteobacteria_Pseudomonadales__Cluster560
Proteobacteria_Rhodospirillales__Cluster132
Proteobacteria_Rhodospirillales__Cluster145
Proteobacteria_Rhodospirillales__Cluster150
Proteobacteria_Rhodospirillales__Cluster214
Proteobacteria_Rhodospirillales__Cluster219
Proteobacteria_Rhodospirillales__Cluster508
Proteobacteria_Rhodospirillales__Cluster561
Proteobacteria_Rhodospirillales__Cluster58 *
Proteobacteria_Rickettsiales__Cluster213
Proteobacteria_SMXQ01__Cluster198
Proteobacteria_Sphingomonadales__Cluster110
Proteobacteria_Sphingomonadales__Cluster114 *
Proteobacteria_Sphingomonadales__Cluster190
Proteobacteria_UBA4486__Cluster166
Proteobacteria_UBA4575__Cluster116
Proteobacteria_UBA9214__Cluster534
Thermoproteota_Nitrososphaerales__Cluster565
Verrucomicrobiota_Chthoniobacterales__Cluster196
Verrucomicrobiota_Opitutales__Cluster195
Verrucomicrobiota_Opitutales__Cluster454
Verrucomicrobiota_Opitutales__Cluster549
Verrucomicrobiota_Verrucomicrobiales__Cluster104
Verrucomicrobiota_Verrucomicrobiales__Cluster129
Verrucomicrobiota_Verrucomicrobiales__Cluster131
max completeness
number of MAGs
thiE
thiG
thiC
thiB
thiT
ThiPerm
thiV
cytX
omr1
thiY
thiM
TenA
1
0.8
0.6
0.4
0.2
0
max completeness
100
60
number of MAGs
1
2
3
4
5
6
7
8
9
13
14
15
17
18
19
20

Figure S12: Clusters possessing *thiC*, which codes for synthesis of pyrimidine synthase. Heatmap shows presence (dark boxes) or absence (gray boxes) of genes across clusters. The gradient of the dark boxes indicates the percentage of MAGs containing the protein. Number of MAGs and maximum estimated completeness of the MAGs are given on the left side. Taxonomy is shown for each cluster on phylum, order and genus levels. Relative abundance of clusters marked with an * shown in Supplementary Figure S7.

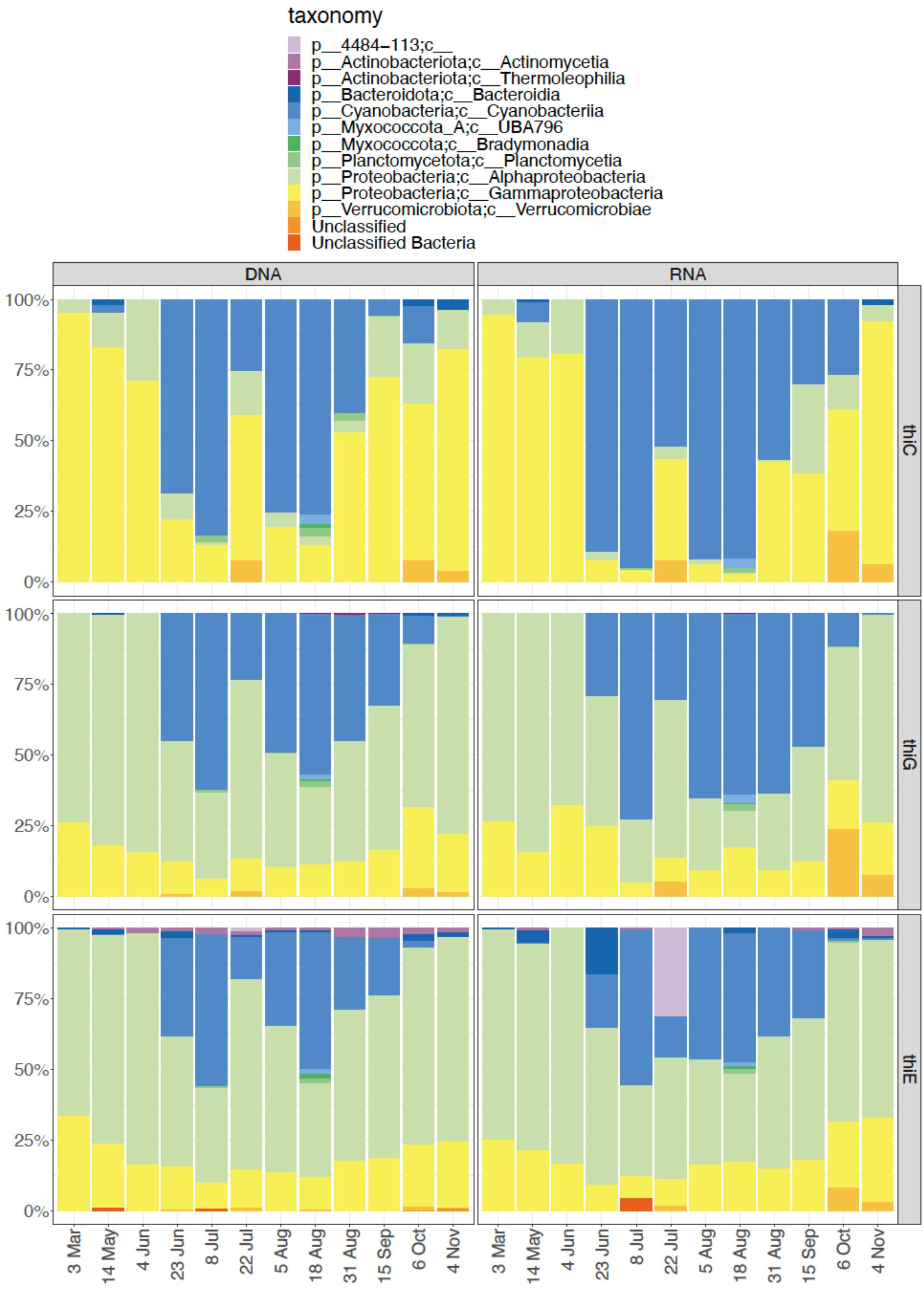

Figure S13: Taxonomic classification of *thiC*, *thiG* and *thiE* genes and transcripts from genomic bins, counts scaled to 100%.

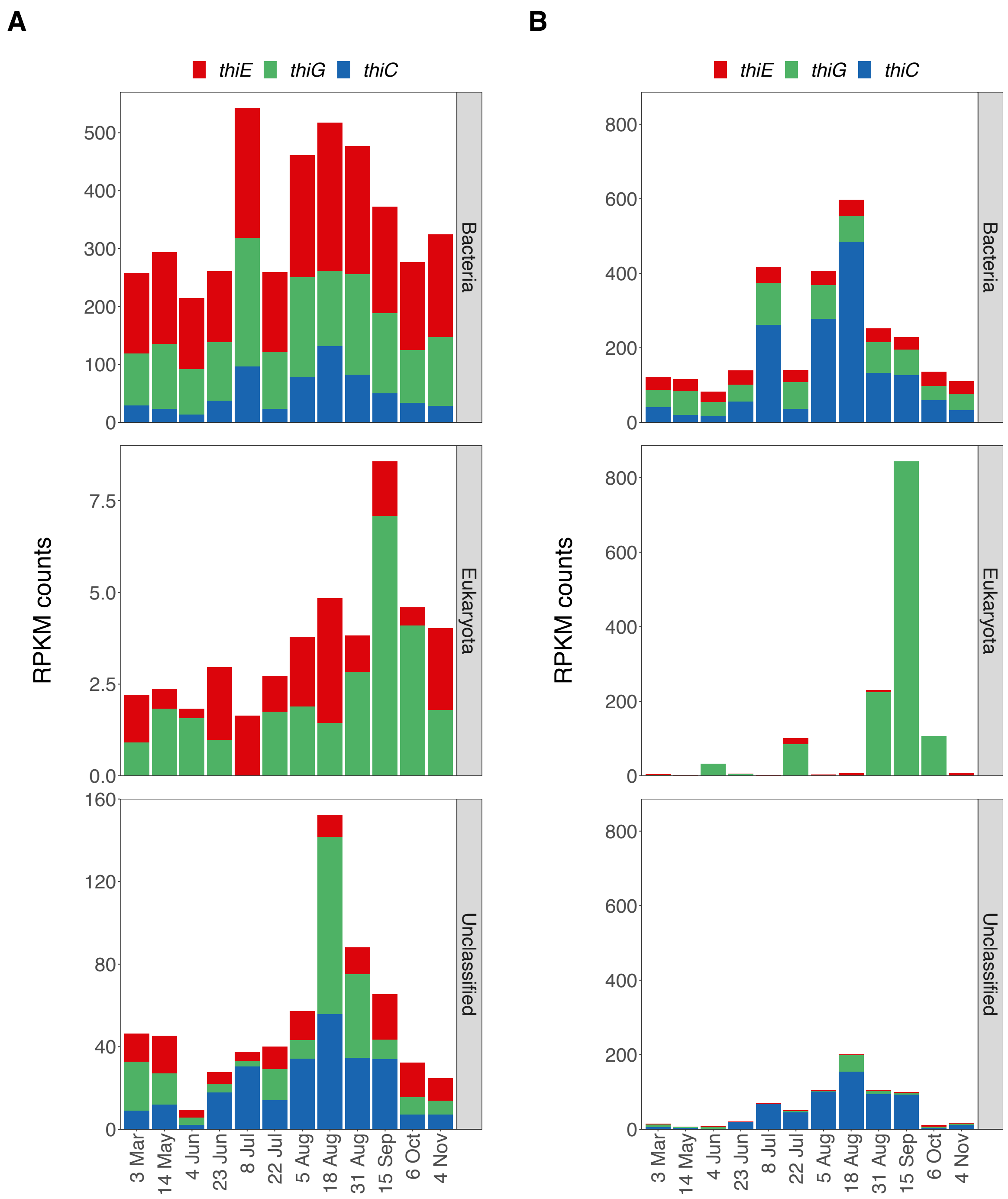

Figure S14: Taxonomic classification of *thiC*, *thiG* and *thiE* genes (**A**) and transcripts (**B**) from assemblies. Counts are Reads Per Kilobase Million (RPKM) normalized.

Supplementary Tables

Table S1: Compound specific LC-MS parameters. Mass over charge (m/z) values and the collision energy (CE) in parenthesis are provided for each parent and the respective products used for quantification of the compound. The limit of detection (LOD) is calculated as three times the variation of the quality control sample. Limit of quantification (LOQ) is calculated as ten times the variation of the quality control sample. LOD and LOQ are given in pM. * values between LOD and LOQ were verified by the batch per batch method.

Compound					Particulate Fraction			Dissolved Fraction		
	precursor m/z	product m/z (CE)	product m/z (CE)	product m/z (CE)	LOD (pM)	LOQ (pM)	Comment	LOD (pM)	LOQ (pM)	Comment
Thiamin	265.2	122.3 (16)	144.2 (16)		0.30	0.99	quantified *	6.96	23.2	quantified *
HET, 4-methyl-5-thiazoleethanol	144.1	113.2 (24)	112.2 (32)	71.3 (36)	0.01	0.03	quantified *	1.06	3.54	quantified
HMP, 4-amino-5-hydroxymethyl-2-methylpyrimidine	140.1	122.2 (12)	54.3 (19)		2.04	6.79	quantified *	10.9	36.2	quantified *
FAMP, N-formyl-4-amino-5-aminomethyl-2-methylpyrimidine	167.1	122.1 (17)	54.1 (33)		0.18	0.60	quantified *	10.4	34.8	quantified *
cHET, 5-(2-hyrdoxyethyl)-4-methyl-1,3-thiazole-2-carboxilic acid	188.1	152.2 (22)	140.2 (21)		0.82	2.72	not detected, < LOD	11.0	36.8	not detected, < LOD
AmMP, 4-amino-5-aminomethyl-2-methylpyrimidine	139.1	122.2 (13)	54.1 (19)		14.6	48.6	trace detected, < LOQ			
TMP, thiamin monophosphate	345.2	122.1 (22)	224 (26)				not detected	19.4	64.7	not detected, < LOD, quantified in a few samples > LOD *

Table S2: Thermal conditions for PCR amplification of 16S and 18S rRNA genes using the 5151F-Y/926R primer set.

Step	Temperature	Duration	Cycles
Initial denaturation	95	3 min	1
Denaturation	95	45 sec	30
Annealing	50	45 sec	30
Extension	72	90 sec	30
Final extension	72	5 min	1

Table S3: Overview of the 17 metagenomic and 12 metatranscriptomic samples generated. The additional metagenomic samples were processes the same way, included in co-assemblies (Supplementary Table S4) and bins used in the genomic analysis.

			metagenome		metatranscriptome	
Date of sampling	***Sample name***	***note***	***Mreads***	***≥Q30***	***Mreads***	***Mreads after QC***
03 March 2020	R1-A		39.38	92.05	87.8	40.0
14 May 2020	R2-A		59.92	90.97	123.7	38.8
04 June 2020	R3-A		64.63	91.24	95.3	38.4
23 June 2020	R4-A		61.17	91.25	167.2	32
08 July 2020	R5-A		52.49	91.14	276.7	101.7
22 July 2020	R6-A		61.7	92.18	185.4	71.7
05 August 2020	R7-A		67.85	91.98	95.6	46.7
18 August 2020	R8-A		63.29	92.2	86	39.8
31 August 2020	R9-A		73.78	92.11	83.7	37.3
15 September 2020	R10-A		75.57	91.21	97.8	39.3
06 October 2020	R11-A		82.98	91.74	99.7	43.1
04 November 2020	R12-A		110.41	91.69	95.9	46.6
18 August 2020	R8-II-B1	additional sample from nutrient amendment experiment (Supplementary Methods 1)	105.71	91.78		
18 August 2020	R8-II-Pre	additional sample from nutrient amendment experiment (Supplementary Methods 1)	81.81	91.54		
18 August 2020	R8-II-con	additional sample from nutrient amendment experiment (Supplementary Methods 1)	82.38	91.71		
22 July 2020	R6-D	additional sample	145.58	92.61		
22 July 2020	R6-E	additional sample	77.09	91.64		

Table S4: Statistics for the 20 assemblies generated with MEGAHIT. Co-assemblies were run to improve read coverage and binning (to obtain bins for less abundant taxa).

Date of sampling	*Assembly name*	*note*	*contigs*	*total_size_bp*	*min_length*	*max_length*	*avg_length*	*median_length*	*N50_length*	*N90_length*	*% metatranscriptomic reads aligned*
03 March 2020	R1-A		196044	342142503	300	388111	1745	762	3439	612	63.8
14 May 2020	R2-A		225718	418406789	300	400427	1854	798	3673	641	38.6
04 June 2020	R3-A		334841	511722897	300	497215	1528	680	2976	550	44.7
23 June 2020	R4-A		256478	455285502	300	460745	1775	749	3679	605	35.3
08 July 2020	R5-A		355087	455127204	300	355393	1282	693	1682	535	47.0
22 July 2020	R6-A		242452	423908534	300	381202	1748	769	3317	613	43.5
05 August 2020	R7-A		267327	489801182	300	460254	1832	798	3589	637	53.4
18 August 2020	R8-A		364612	587492078	300	563515	1611	749	2769	590	67.3
31 August 2020	R9-A		338180	505523338	300	364166	1495	714	2375	561	56.0
15 September 2020	R10-A		313350	521438650	300	362933	1664	741	3051	591	64.1
06 October 2020	R11-A		383578	628895000	300	435342	1640	728	3049	584	60.2
04 November 2020	R12-A		457430	772754342	300	494541	1689	773	2922	608	66.4
18 August 2020	R8-II-B1	nutrient amendment experiment	483993	785499457	300	497623	1623	757	2737	595	
18 August 2020	R8-II-Pre	nutrient amendment experiment	422296	680259249	300	515834	1611	749	2728	591	
18 August 2020	R8-II-con	nutrient amendment experiment	431567	681511960	300	497679	1579	739	2654	584	
22 July 2020	R6-D	additional sample	476508	697607794	300	408782	1464	738	2159	567	
22 July 2020	R6-E	additional sample	422883	636640733	300	492411	1505	761	2245	583	
**co-assembly**	R	all samples	2121316	3458169683	300	675765	1630	792	2668	607	
**co-assembly**	R6	three samples from 4 June 2020	627888	1074178794	300	564763	1711	783	3076	613	
**co-assembly**	R8	sample from 18 August and three samples form incbuation experiment	977328	1563708104	300	664698	1600	791	2477	605	

Table S5: Hmm protein models used in the surveys for protein sequences attributed to B1 synthesis, transport or salvage. na: not applicable

Protein Target	***Protein Model***	***full-score cut-off***	***Evaluation***
ThiC	PF01964.17, TIGR00190	na	both models hit the same ORF, TIGERFAM model ususally scores better, all scores very high and can be considered true positives
ThiG	PF05690.13	> 48	
ThiE	PF02581.16, TIGR00693	> 40	Usually protein models hit the same ORF, sometimes Pfam model gets a few additional hits that seem to be true positives.
ThiB	TIGR01276, TIGR01254	> 65	Many sequences were false positive hits and related to iron uptake.
ThiT	TIGR02357	na	Hits can be considered true positives, TDP riboswitch in proximity. Screened sequences originated from Firmicutes taxa, which have been previously described to encode ThiT.
ThiY	custom - Paerl et al. 2018 PNAS	> 100	Many sequences that clustered with the reference sequences in the phylogeny were in proximity to a TDP riboswitch and/or other genes related to B1.
ThiV	custom - Paerl et al. 2018 PNAS	> 100	Clear clustering of sequences observed next to references sequences in phylogeny and mostly with a TDP riboswitch in proximity.
TenA	TIGR04306, (PF03070.15)		Pfam model is too broad and gives mostly false positives, ussually true positives are picked up by the TIGERFAM model with a high score. The only exception observed is for the Pelagibacterales genus IMCC9063, which gets a higher score with the Pfam model. Hits include both protein forms TenA_C and TenA_E.
ThiM	PF02110.14, TIGR00694	> 50	
PnuC	TIGR01528	na	Hits can be considered true positives, most sequences screen had the known AA residues interacting with B1. About half of the sequences screened had TDP riboswitch in proxmity to the gene.
YkoF	PF07615.10	na	Hits can be considered true positives, most sequences screened had TDP riboswitch in proxmity to the gene.
CytX	TIGR02358	na	Hits are usually not very strong (e-value and full-score)
ThiPerm	PF02133.14	na	Hits are usually not very strong (e-value and full-score)

Table S6: Single-copy marker genes used for normalisation of gene abundance of metatranscriptomes according to Salazar et al. 2019. For KO profiles 10 KOs were used and for pfam-hmmsearch output 21 Pfam models were used.

marker ID	***description***
K06942	ychF; ribosome-binding ATPase
K01889	FARSA, pheS; phenylalanyl-tRNA synthetase alpha chain [EC:6.1.1.20]
K01887	RARS, argS; arginyl-tRNA synthetase [EC:6.1.1.19]
K01875	SARS, serS; seryl-tRNA synthetase [EC:6.1.1.11]
K01883	CARS, cysS; cysteinyl-tRNA synthetase [EC:6.1.1.16]
K01869	LARS, leuS; leucyl-tRNA synthetase [EC:6.1.1.4]
K01873	VARS, valS; valyl-tRNA synthetase [EC:6.1.1.9]
K01409	OSGEP, KAE1, QRI7; N6-L-threonylcarbamoyladenine synthase [EC:2.3.1.234]
K03106	SRP54, ffh; signal recognition particle subunit SRP54 [EC:3.6.5.4]
K03110	ftsY; fused signal recognition particle receptor
PF00133.21	tRNA synthetases class I (I, L, M and V)
PF00448.21	SRP54-type protein, GTPase domain
PF00587.24	tRNA synthetase class II core domain (G, H, P, S and T)
PF00750.18	tRNA synthetases class I (R)
PF00814.24	tRNA N6-adenosine threonylcarbamoyltransferase
PF01406.18	tRNA synthetases class I (C) catalytic domain
PF01409.19	tRNA synthetases class II core domain (F)
PF01926.22	50S ribosome-binding GTPase
PF02403.21	Seryl-tRNA synthetase N-terminal domain
PF02881.18	SRP54-type protein, helical bundle domain
PF02912.17	Aminoacyl tRNA synthetase class II, N-terminal domain
PF03483.16	B3/B4 tRNA-binding domain
PF03484.14	tRNA synthetase B5 domain
PF03485.15	Arginyl tRNA synthetase N terminal domain
PF05746.14	DALR anticodon binding domain
PF06071.12	YchF-GTPase C terminal protein domain
PF08264.12	Aminoacyl tRNA synthetase
PF09190.10	Aminoacyl tRNA synthetase
PF09334.10	tRNA synthetases class I (M)
PF10458.8	Valyl tRNA synthetase tRNA binding arm
PF13603.5	Leucyl-tRNA synthetase, Domain 2

Table S7: Select Kendall correlations between environmental parameters, B1 and vitamer concentrations, relative abundances of taxa, gene and transcript counts. ns: not significant; asterisk indicate p-value levels: *: p < 0.05, **: p ≤ 0.01, ***: p ≤ 0.001, ****: p ≤ 0.0001

Variables	*Kendalls tau*	*p-value*	*significance level*
Secchi disk depth - dissolved HET	0.54	0.01560	*
Chl a - dissolved B1			ns
Chl a - N:P ratio	-0.69	0.00228	**
Chl a - total pyrimidines	0.45	0.04470	*
temperature - particulate FAMP	0.73	0.00050	***
temperature - dissolved FAMP	0.55	0.01380	*
temperature - dissolved HMP	0.73	0.00050	***
temperature - dissolved B1	0.48	0.03110	*
bacterial abundance - dissolved B1			ns
dissolved B1 - dissolved HMP	0.64	0.00318	**
dissolved HMP - dissolved HET	-0.45	0.04470	*
dissolved HMP - dissolved FAMP	0.52	0.02100	*
dissolved HMP - particulate FAMP	0.58	0.00876	**
PCC-6307 relative abundance - total B1 pool	0.45	0.04470	*
PCC-6307 relative abundance - PBV B1	0.64	0.00318	**
PCC-6307 relative abundance - thiC gene counts	0.65	0.00318	**
PCC-6307 relative abundance - Chl a	0.55	0.01380	*
PCC-6307 relative abundance - temperature	0.58	0.00876	**
Pelagibacterales relative abundance - Chl a	-0.45	0.04470	*
Burkholderiales relative abundance - temperature	-0.45	0.04470	*
Pelagibacterales relative abundance - temperature	-0.48	0.03110	*
Cyanobacteria relative abundance - temperature	0.52	0.02100	*
Proteobacteria relative abundance - temperature	-0.52	0.02100	*
total B1 pool - temperature	0.7	0.00097	***
relative abundance thiC containing clusters - thiC RNA	0.73	0.00050	***
Cyanobacteria relative abundance - thiC RNA	0.67	0.00180	**
Cyanobacteria relative abundance - Cyanobacteria thiC gene counts	0.69	0.00198	**
Cyanobacteria relative abundance - thiC gene counts	0.71	0.00153	**
Cyanobacteria relative abundance - particulate B1	0.58	0.00876	**
total particulate B1 pool - POC	0.6	0.01670	*
total particulate B1 pool - production	0.52	0.02100	*
particulate HMP - eukaryotic plankton biomass	0.58	0.00876	**
particulate HMP - Ciliophora biomass	0.73	0.00050	***
particulate HMP - Dinoflagellata biomass	0.48	0.03570	*
dissolved HET - Ciliophora biomass	-0.48	0.03110	*

total particulate B1 and vitamers - Ciliophora biomass	0.48	0.03110	*
particulate B1 - Chlorophyta biomass			ns
particulate B1 - Ciliophora biomass			ns
particulate B1 - Cryptophyta biomass			ns
particulate B1 - Dinoflagellata biomass			ns
particulate B1 - Euglenophyta biomass			ns
particulate B1 - Diatoms_Coscinodiscophyceae biomass			ns
particulate B1 - Ochrophyta.Dictyochophyceae biomass			ns
particulate B1 - Flagellates biomass			ns
particulate HET - Chlorophyta biomass			ns
particulate HET - Ciliophora biomass			ns
particulate HET - Cryptophyta biomass			ns
particulate HET - Dinoflagellata biomass			ns
particulate HET - Euglenophyta biomass			ns
particulate HET - Diatoms_Coscinodiscophyceae biomass			ns
particulate HET - Ochrophyta.Dictyochophyceae biomass			ns
particulate HET - Flagellates biomass			ns
particulate HMP - Chlorophyta biomass			ns
particulate HMP - Cryptophyta biomass			ns
particulate HMP - Euglenophyta biomass			ns
particulate HMP - Diatoms_Coscinodiscophyceae biomass			ns
particulate HMP - Ochrophyta.Dictyochophyceae biomass			ns
particulate HMP - Flagellates biomass			ns
particulate FAMP - Chlorophyta biomass			ns
particulate FAMP - Ciliophora biomass			ns
particulate FAMP - Cryptophyta biomass			ns
particulate FAMP - Dinoflagellata biomass			ns
particulate FAMP - Euglenophyta biomass			ns
particulate FAMP - Diatoms_Coscinodiscophyceae biomass			ns
particulate FAMP - Ochrophyta.Dictyochophyceae biomass			ns
particulate FAMP - Flagellates biomass			ns
dissolved B1 - Chlorophyta biomass			ns
dissolved B1 - Ciliophora biomass			ns
dissolved B1 - Cryptophyta biomass			ns
dissolved B1 - Dinoflagellata biomass			ns
dissolved B1 - Euglenophyta biomass			ns
dissolved B1 - Diatoms_Coscinodiscophyceae biomass			ns
dissolved B1 - Ochrophyta.Dictyochophyceae biomass			ns
dissolved B1 - Flagellates biomass			ns
dissolved HET - Chlorophyta biomass			ns
dissolved HET - Cryptophyta biomass			ns
dissolved HET - Dinoflagellata biomass			ns
dissolved HET - Euglenophyta biomass			ns

dissolved HET - Diatoms_Coscinodiscophyceae biomass	ns
dissolved HET - Ochrophyta.Dictyochophyceae biomass	ns
dissolved HET - Flagellates biomass	ns
dissolved HMP - Chlorophyta biomass	ns
dissolved HMP - Ciliophora biomass	ns
dissolved HMP - Cryptophyta biomass	ns
dissolved HMP - Dinoflagellata biomass	ns
dissolved HMP - Euglenophyta biomass	ns
dissolved HMP - Diatoms_Coscinodiscophyceae biomass	ns
dissolved HMP - Ochrophyta.Dictyochophyceae biomass	ns
dissolved HMP - Flagellates biomass	ns
dissolved FAMP - Chlorophyta biomass	ns
dissolved FAMP - Ciliophora biomass	ns
dissolved FAMP - Cryptophyta biomass	ns
dissolved FAMP - Dinoflagellata biomass	ns
dissolved FAMP - Euglenophyta biomass	ns
dissolved FAMP - Diatoms_Coscinodiscophyceae biomass	ns
dissolved FAMP - Ochrophyta.Dictyochophyceae biomass	ns
dissolved FAMP - Flagellates biomass	ns
total particulate B1 and vitamers - Chlorophyta biomass	ns
total particulate B1 and vitamers - Cryptophyta biomass	ns
total particulate B1 and vitamers - Dinoflagellata biomass	ns
total particulate B1 and vitamers - Euglenophyta biomass	ns
total particulate B1 and vitamers - Diatoms_Coscinodiscophyceae biomass	ns
total particulate B1 and vitamers - Ochrophyta.Dictyochophyceae biomass	ns
total particulate B1 and vitamers - Flagellates biomass	ns
total dissolved B1 and vitamers - Chlorophyta biomass	ns
total dissolved B1 and vitamers - Ciliophora biomass	ns
total dissolved B1 and vitamers - Cryptophyta biomass	ns
total dissolved B1 and vitamers - Dinoflagellata biomass	ns
total dissolved B1 and vitamers - Euglenophyta biomass	ns
total dissolved B1 and vitamers - Diatoms_Coscinodiscophyceae biomass	ns
total dissolved B1 and vitamers - Ochrophyta.Dictyochophyceae biomass	ns
total dissolved B1 and vitamers - Flagellates biomass	ns
thiC gene counts - dissolved B1	ns
thiC gene counts - dissolved HET	ns
thiC gene counts - dissolved HMP	ns
thiC gene counts - dissolved FAMP	ns
thiC gene counts - particulate B1	ns
thiC gene counts - particulate HET	ns
thiC gene counts - particulate HMP	ns
thiC gene counts - particulate FAMP	ns
thiC transcript counts - dissolved B1	ns

thiC transcript counts - dissolved HET			ns
thiC transcript counts - dissolved HMP			ns
thiC transcript counts - dissolved FAMP			ns
thiC transcript counts - particulate B1			ns
thiC transcript counts - particulate HET			ns
thiC transcript counts - particulate HMP			ns
thiC transcript counts - particulate FAMP			ns
thiG gene counts - dissolved B1			ns
thiG gene counts - dissolved HET			ns
thiG gene counts - dissolved HMP			ns
thiG gene counts - dissolved FAMP			ns
thiG gene counts - particulate B1			ns
thiG gene counts - particulate HET			ns
thiG gene counts - particulate HMP			ns
thiG gene counts - particulate FAMP			ns
thiG transcript counts - dissolved B1			ns
thiG transcript counts - dissolved HET			ns
thiG transcript counts - dissolved HMP			ns
thiG transcript counts - dissolved FAMP			ns
thiG transcript counts - particulate B1			ns
thiG transcript counts - particulate HET			ns
thiG transcript counts - particulate HMP			ns
thiG transcript counts - particulate FAMP			ns
thiE gene counts - dissolved B1			ns
thiE gene counts - dissolved HET			ns
thiE gene counts - dissolved HMP			ns
thiE gene counts - dissolved FAMP			ns
thiE gene counts - particulate B1			ns
thiE gene counts - particulate HET	-0.47	0.0375	*
thiE gene counts - particulate HMP			ns
thiE gene counts - particulate FAMP			ns
thiE transcript counts - dissolved B1	-0.45	0.04470	*
thiE transcript counts - dissolved HET			ns
thiE transcript counts - dissolved HMP			ns
thiE transcript counts - dissolved FAMP			ns
thiE transcript counts - particulate B1			
thiE transcript counts - particulate HET			
thiE transcript counts - particulate HMP			
thiE transcript counts - particulate FAMP			

Table S8: Maximum cell yields of *V. anguillarium* PF430-3 ΔthiE in bioassay of RF water (1:10 diluted) from 4 June 2020. Yields from no B1 addition (0 pM) and added B1 (5, 10, 25, 50, 75 pM) were used to generate an internal standard curve (Figure S3, Equation) and ultimately calculate the bioavailable dissolved B1 concentration, based on PF430-3 ΔthiE yields per supplemented B1, see Methods in the main text. No growth was observed in the negative controls on B1-deplete medium.

pM B1 added	*Replicate 1*	*Replicate 2*	*Replicate 3*	*Replicate 4*
0	2.17E+06	2.45E+06	2.78E+06	2.24E+06
5	7.23E+05	1.76E+06	1.67E+06	9.61E+05
10	1.82E+06	8.31E+05	2.47E+06	3.16E+06
25	3.06E+06	6.31E+06	5.78E+06	3.32E+06
50	1.26E+07	1.19E+07	1.56E+07	1.22E+07
75	1.59E+07	1.81E+07	1.87E+07	2.06E+07
calculated pM B1 in RF	116.42	127.25	139.69	118.97

Table S9: Biomass of small eukaryotes and bacteria. Small eukaryotes were identified by microscopy and taxa specific conversion factors applied [45]. Bacterial cell abundance was determined by flow cytometry and a conversion factor of 20 fg C per cell was applied [5].

Date of sampling	*small eukayotic biomass (µgC/L)*	*Bacterial biomass (µgC/L)*
03 March 2020	7.30	146.80
14 May 2020	58.80	321.80
04 June 2020	128.00	141.00
23 June 2020	72.90	273.60
08 July 2020	116.60	192.60
22 July 2020	54.50	178.00
05 August 2020	50.80	324.00
18 August 2020	53.60	214.40
31 August 2020	8.50	208.80
15 September 2020	56.80	204.80
06 October 2020	71.40	202.20
04 November 2020	1.20	115.00

References

1. Paerl RW, Sundh J, Tan D, Svenningsen SL, Hylander S, Pinhassi J, et al. Prevalent reliance of bacterioplankton on exogenous vitamin B1 and precursor availability. *Proc Natl Acad Sci* 2018; **115**: E10447–E10456.
2. Okbamichael M, Sañudo-Wilhelmy SA. Direct determination of vitamin B 1 in seawater by solid-phase extraction and high-performance liquid chromatography quantification. *Limnol Oceanogr Methods* 2005; **3**: 241–246.
3. Smith DC, Azam F. A simple, economical method for measuring bacterial protein synthesis rates in seawater using 3H-leucine. *Mar Microb Food Webs* 1992; **6**: 107–114.
4. Carlson CA, Ducklow HW, Sleeter TD. Stocks and dynamics of bacterioplankton in the northwestern Sargasso Sea. *Deep Sea Res Part II Top Stud Oceanogr* 1996; **43**: 491–515.
5. Lee S, Fuhrman JA. Relationships between Biovolume and Biomass of Naturally Derived Marine Bacterioplankton. *Appl Environ Microbiol* 1987; **53**: 1298–1303.
6. Heal KR, Qin W, Ribalet F, Bertagnolli AD, Coyote-Maestas W, Hmelo LR, et al. Two distinct pools of B_{12} analogs reveal community interdependencies in the ocean. *Proc Natl Acad Sci* 2017; **114**: 364–369.
7. Boysen AK, Heal KR, Carlson LT, Ingalls AE. Best-Matched Internal Standard Normalization in Liquid Chromatography-Mass Spectrometry Metabolomics Applied to Environmental Samples. *Anal Chem* 2018; **90**: 1363–1369.
8. Suffridge CP, Bolaños LM, Bergauer K, Worden AZ, Morré J, Behrenfeld MJ, et al. Exploring Vitamin B1 Cycling and Its Connections to the Microbial Community in the North Atlantic Ocean. *Front Mar Sci* 2020; **7**: 1–16.
9. Suffridge C, Cutter L, Sañudo-Wilhelmy SAS. A New Analytical Method for Direct Measurement of Particulate and Dissolved B-vitamins and Their Congeners in Seawater. *Front Mar Sci* 2017; **4**: 1–11.
10. Suffridge CP, Gómez-Consarnau L, Monteverde DR, Cutter L, Arístegui J, Alvarez-Salgado XA, et al. B Vitamins and Their Congeners as Potential Drivers of Microbial Community Composition in an Oligotrophic Marine Ecosystem. *J Geophys Res Biogeosciences* 2018; **123**: 2890–2907.

11. Heal KR, Carlson LT ruxa., Devol AH, Armbrust EV, Moffett JW, Stahl DA, et al. Determination of four forms of vitamin B 12 and other B vitamins in seawater by liquid chromatography/tandem mass spectrometry. *Rapid Commun Mass Spectrom* 2014; **28**: 2398–2404.
12. Stocker R. Marine Microbes See a Sea of Gradients. *Science (80-)* 2012; **338**: 628–633.
13. Sañudo-Wilhelmy SA, Cutter LS, Durazo R, Smail EA, Gómez-Consarnau L, Webb EA, et al. Multiple B-vitamin depletion in large areas of the coastal ocean. *Proc Natl Acad Sci* 2012; **109**: 14041–14045.
14. Johnson WM, Kido Soule MC, Kujawinski EB. Extraction efficiency and quantification of dissolved metabolites in targeted marine metabolomics. *Limnol Oceanogr Methods* 2017; **15**: 417–428.
15. Sacks JS, Heal KR, Boysen AK, Carlson LT, Ingalls AE. Quantification of dissolved metabolites in environmental samples through cation-exchange solid-phase extraction paired with liquid chromatography–mass spectrometry. *Limnol Oceanogr Methods* 2022; **20**: 683–700.
16. Yeh Y-C, Fuhrman JA. Contrasting diversity patterns of prokaryotes and protists over time and depth at the San-Pedro Ocean Time series. *ISME Commun* 2022; **2**: 1–12.
17. McNichol J, Berube PM, Biller SJ, Fuhrman JA. Evaluating and Improving Small Subunit rRNA PCR Primer Coverage for Bacteria, Archaea, and Eukaryotes Using Metagenomes from Global Ocean Surveys. *mSystems* 2021; **6**.
18. Martin M. Cutadapt removes adapter sequences from high-throughput sequencing reads. *EMBnet.journal* 2011; **17**: 10.
19. Boratyn GM, Thierry-Mieg J, Thierry-Mieg D, Busby B, Madden TL. Magic-BLAST, an accurate RNA-seq aligner for long and short reads. *BMC Bioinformatics* 2019; **20**: 405.
20. Guillou L, Bachar D, Audic S, Bass D, Berney C, Bittner L, et al. The Protist Ribosomal Reference database (PR2): a catalog of unicellular eukaryote Small Sub-Unit rRNA sequences with curated taxonomy. *Nucleic Acids Res* 2012; **41**: D597–D604.
21. McMurdie PJ, Holmes S. phyloseq: An R Package for Reproducible Interactive Analysis and Graphics of Microbiome Census Data. *PLoS One* 2013; **8**: e61217.
22. Li D, Luo R, Liu C-M, Leung C-M, Ting H-F, Sadakane K, et al. MEGAHIT v1.0: A fast and scalable metagenome assembler driven by advanced methodologies and community practices. *Methods* 2016; **102**: 3–11.

23. Hyatt D, Chen G-L, LoCascio PF, Land ML, Larimer FW, Hauser LJ. Prodigal: prokaryotic gene recognition and translation initiation site identification. *BMC Bioinformatics* 2010; **11**: 119.
24. Nawrocki EP, Eddy SR. Infernal 1.1: 100-fold faster RNA homology searches. *Bioinformatics* 2013; **29**: 2933–2935.
25. Huerta-Cepas J, Forslund K, Coelho LP, Szklarczyk D, Jensen LJ, von Mering C, et al. Fast Genome-Wide Functional Annotation through Orthology Assignment by eggNOG-Mapper. *Mol Biol Evol* 2017; **34**: 2115–2122.
26. Mistry J, Chuguransky S, Williams L, Qureshi M, Salazar GA, Sonnhammer ELL, et al. Pfam: The protein families database in 2021. *Nucleic Acids Res* 2021; **49**: D412–D419.
27. Haft DH, Selengut JD, Richter RA, Harkins D, Basu MK, Beck E. TIGRFAMs and Genome Properties in 2013. *Nucleic Acids Res* 2012; **41**: D387–D395.
28. Pierce NT, Irber L, Reiter T, Brooks P, Brown CT. Large-scale sequence comparisons with sourmash. *F1000Research* 2019; **8**: 1006.
29. Miranda-Ríos J, Navarro M, Soberón M. A conserved RNA structure (thi box) is involved in regulation of thiamin biosynthetic gene expression in bacteria. *Proc Natl Acad Sci* 2001; **98**: 9736–9741.
30. Rodionov DA, Vitreschak AG, Mironov AA, Gelfand MS. Comparative Genomics of Thiamin Biosynthesis in Procaryotes. *J Biol Chem* 2002; **277**: 48949–48959.
31. Kang DD, Li F, Kirton E, Thomas A, Egan R, An H, et al. MetaBAT 2: an adaptive binning algorithm for robust and efficient genome reconstruction from metagenome assemblies. *PeerJ* 2019; **7**: e7359.
32. Alneberg J, Bjarnason BS, De Bruijn I, Schirmer M, Quick J, Ijaz UZ, et al. Binning metagenomic contigs by coverage and composition. *Nat Methods* 2014; **11**: 1144–1146.
33. Parks DH, Imelfort M, Skennerton CT, Hugenholtz P, Tyson GW. CheckM: assessing the quality of microbial genomes recovered from isolates, single cells, and metagenomes. *Genome Res* 2015; **25**: 1043–1055.
34. Chaumeil P-A, Mussig AJ, Hugenholtz P, Parks DH. GTDB-Tk: a toolkit to classify genomes with the Genome Taxonomy Database. *Bioinformatics* 2019; **36**: 1925–1927.
35. Jain C, Rodriguez-R LM, Phillippy AM, Konstantinidis KT, Aluru S. High throughput ANI analysis of 90K prokaryotic genomes reveals clear species boundaries. *Nat Commun*

2018; **9**: 5114.

36. Bowers RM, Kyrpides NC, Stepanauskas R, Harmon-Smith M, Doud D, Reddy TBK, et al. Minimum information about a single amplified genome (MISAG) and a metagenome-assembled genome (MIMAG) of bacteria and archaea. *Nat Biotechnol* 2017; **35**: 725–731.
37. Parks DH, Chuvochina M, Chaumeil P, Rinke C, Mussig AJ, Hugenholtz P. A complete domain-to-species taxonomy for Bacteria and Archaea. *Nat Biotechnol* 2020; **38**: 1079–1086.
38. Kopylova E, Noé L, Touzet H. SortMeRNA: fast and accurate filtering of ribosomal RNAs in metatranscriptomic data. *Bioinformatics* 2012; **28**: 3211–3217.
39. Langmead B, Salzberg SL. Fast gapped-read alignment with Bowtie 2. *Nat Methods* 2012; **9**: 357–359.
40. Salazar G, Paoli L, Alberti A, Huerta-Cepas J, Ruscheweyh H-JJ, Cuenca M, et al. Gene Expression Changes and Community Turnover Differentially Shape the Global Ocean Metatranscriptome. *Cell* 2019; **179**: 1068-1083.e21.
41. Altschul SF, Gish W, Miller W, Myers EW, Lipman DJ. Basic local alignment search tool. *J Mol Biol* 1990; **215**: 403–410.
42. O'Leary NA, Wright MW, Brister JR, Ciufo S, Haddad D, McVeigh R, et al. Reference sequence (RefSeq) database at NCBI: current status, taxonomic expansion, and functional annotation. *Nucleic Acids Res* 2016; **44**: D733–D745.
43. Bateman A, Martin M-J, Orchard S, Magrane M, Ahmad S, Alpi E, et al. UniProt: the Universal Protein Knowledgebase in 2023. *Nucleic Acids Res* 2023; **51**: D523–D531.
44. Dataforsyningen. GeoDanmark grunddata: Natur- og Friluftskort 2022. 2023. Styrelsen for Dataforsyning og Infrastruktur (SDFI), WMS Service.
45. Strathmann RR. Estimating the Organic Carbon Content of Phytoplankton From Cell Volume or Plasma Volume. *Limnol Oceanogr* 1967; **12**: 411–418.
